# Virulence on Pm4 kinase-based resistance is determined by two divergent wheat powdery mildew effectors

**DOI:** 10.1038/s41477-025-02180-w

**Published:** 2026-01-12

**Authors:** Zoe Bernasconi, Aline G. Herger, Maria Del Pilar Caro, Lukas Kunz, Marion C. Müller, Ursin Stirnemann, Megan A. Outram, Victoria Widrig, Matthias Neidhart, Jonatan Isaksson, Seraina Schudel, Sebastian Rösli, Thomas Wicker, Kyle W. Bender, Cyril Zipfel, Peter N. Dodds, Melania Figueroa, Javier Sánchez-Martín, Beat Keller

**Affiliations:** 1 Department of Plant and Microbial Biology, University of Zurich, Zurich, Switzerland; 2 Agriculture and Food, Commonwealth Scientific and Industrial Research Organisation, Canberra, Australian Capital Territory Australia; 3 Department of Microbiology and Genetics, Spanish-Portuguese Agricultural Research Centre, University of Salamanca, Salamanca, Spain; 4 Sainsbury Laboratory, University of East Anglia, Norwich, UK; 5 Present Address: Phytopathology, TUM School of Life Sciences, Technical University of Munich, Freising, Germany

**Keywords:** Genetic association study, Effectors in plant pathology, Biotic, Plant breeding

## Abstract

The wheat resistance gene *Pm4* encodes a kinase fusion protein and has gained particular attention as it confers race-specific resistance against two major wheat pathogens: powdery mildew and blast. Here we describe the identification of AvrPm4, the mildew avirulence effector recognized by Pm4, using UV mutagenesis, and its functional validation in wheat protoplasts. We show that AvrPm4 directly interacts with and is phosphorylated by Pm4. Using genetic association and quantitative trait locus mapping, we further demonstrate that the evasion of *Pm4* resistance by virulent mildew isolates relies on a second fungal component, *SvrPm4*, which suppresses *AvrPm4*-induced cell death. Surprisingly, *SvrPm4* was previously described as *AvrPm1a*. We show that SvrPm4, but not its inactive variant svrPm4, is recognized by the nucleotide-binding leucine-rich repeat immune receptor Pm1a. These multiple roles of a single effector provide a new perspective on fungal (a)virulence proteins and their combinatorial interactions with different types of immune receptors.

## Abstract (web summary)

AvrPm4 is a chimeric powdery mildew effector that is recognized by the wheat kinase fusion resistance protein Pm4. The pathogen can evade Pm4 recognition by expressing the suppressor SvrPm4, an RNase-like effector with multiple roles.

## Main

Wheat yields are severely impacted by pests and pathogens, accounting for over 20% of global losses^1^. Resistance breeding is a key strategy for crop protection and reduction of pathogen-inflicted damage. It often relies on dominant resistance (*R*) genes encoding immune receptors that recognize pathogen-delivered avirulence (Avr) effectors and activate immune responses, usually culminating in cell death^2,3^. While most cloned *R* genes in wheat encode nucleotide-binding leucine-rich repeat (NLR) receptors, kinase fusion proteins (KFPs) are emerging as a distinct class of immune receptors specifically found in cereals^4^. Much of our current understanding of KFPs comes from tandem kinase proteins—a major KFP subclass—which consist of two kinase domains, sometimes with additional domains of unknown function^5,6^. Recent studies have demonstrated that some tandem kinase proteins rely on a helper NLR to trigger effector-induced cell death, as shown for Sr62 in *Aegilops tauschii* and RWT4 in wheat^7,8^.

Beyond tandem kinase proteins, other KFPs, composed of at least one kinase domain and additional domains, have been described in cereals but remain poorly understood^9,10^. Among them, Pm4, a protein containing a serine/threonine kinase, multiple C2 domains and transmembrane regions, is particularly notable: the *Pm4* gene, located on wheat chromosome 2A, encodes two alternative isoforms derived by alternative splicing, Pm4-V1 and Pm4-V2, both required for resistance to the biotrophic pathogen *Blumeria graminis* f. sp. *tritici*^11^. *Pm4* occurs as several alleles (*Pm4a–g*), which encode highly similar proteins. *Pm4a* and *Pm4b* have been studied extensively in near-isogenic backgrounds, where they show partially overlapping race-specific resistance spectra against *B. g. tritici*^11^. Furthermore, recent work has shown that *Pm4* also confers resistance to the hemibiotrophic wheat blast pathogen *Magnaporthe oryzae*^12,13^. In fact, the wheat blast resistance gene *Rmg7* is identical to *Pm4a*, whereas the *Rmg8* resistance gene on wheat chromosome 2B could be assigned to a *Pm4* homoeologue with an identical sequence to that of *Pm4f*^12^. The corresponding wheat blast effector *AVR-Rmg8* was cloned earlier^14^ and is recognized by multiple *Pm4* alleles, including *Pm4a*, *Pm4b* and *Pm4f*^12,13^. The KFP encoded by *Pm4* therefore represents a highly important resistance source in wheat with multiple alleles providing resistance to the obligate biotrophic *B. g. tritici* and the hemibiotrophic wheat blast pathogen simultaneously.

Despite the progress made in describing novel KFPs, a mechanistic understanding of how such proteins induce immunity is yet to be established. Kinase activity in KFPs was suggested to be relevant for Avr effector recognition and/or downstream signalling, a fact further supported by the finding of loss-of-function mutants affected in the kinase domain of KFPs such as Pm4 (ref. ^11^). Moreover, the tandem kinase protein RWT4, encoded by an allele of the powdery mildew resistance gene *Pm24* (refs. ^15,16^), phosphorylates the avirulence but not the virulence variant of the wheat blast effector PWT4, suggesting that the phosphorylation of the Avr effector plays a key role in the resistance mechanism^17^. For the barley stem rust resistance protein RPG1, two fungal proteins work synergistically to induce phosphorylation and trigger a hypersensitive response (HR)^18^. These examples also highlight the importance of direct interaction between KFPs and their recognized effectors. Understanding these mechanisms will require the identification and characterization of Avr effectors that interact with KFPs such as Pm4.

Methods based on genetic association, such as biparental mapping or genome-wide association studies (GWAS), have been widely used to identify *Avr* loci in *Blumeria* and have been combined with cell death assays in heterologous systems such as *Nicotiana benthamiana* and host protoplasts^19–21^. More recently, AvrXpose, an approach based on UV mutagenesis and selection of gain-of-virulence mutants, has also proved effective in identifying genes controlling avirulence in *B. g. tritici*^22^. To date, all known *Avr* genes in *B. g. tritici* encode small (100–150 amino acid residues), secreted effector proteins with a predicted RNase-like structure and trigger a cell death response upon recognition by corresponding wheat NLR immune receptors (reviewed in ref. ^23^). In contrast, no *B. g. tritici* Avr effector recognized by a KFP has been identified so far.

Suppressors of *R*-gene-mediated immunity have been reported across bacterial, oomycete and fungal pathogens^24^. Notable examples from phytopathogenic fungi include the *I* locus in *Melampsora lini*, suppressing L7- and Lx-mediated resistance in flax^25^; *AvrLm4-7* in *Leptosphaeria maculans*, suppressing *Rlm3*- and *Rlm9*-mediated resistance in oilseed rape^26^; and the *SvrPm3* effector in *B. g. tritici*, with the ability to suppress resistance mediated by the wheat NLR Pm3 (refs. ^19,27^). Most suppressors identified so far suppress NLR-mediated immunity via diverse molecular mechanisms including interference with Avr recognition, NLR signalling or NLR homeostasis^24^. In contrast, little is known about the existence or mode of action of suppressor proteins affecting non-NLR resistance proteins such as KFPs.

In this study, we describe the identification of *AvrPm4* from *B. g. tritici* using UV mutagenesis and show that the encoded protein is recognized by the wheat Pm4 resistance protein, resulting in cell death. AvrPm4 is a non-canonical Avr effector that directly interacts with Pm4. Furthermore, using genetic association and quantitative trait locus (QTL) mapping, we describe the identification of *SvrPm4*, a suppressor of *AvrPm4*-induced cell death, and show that *SvrPm4* is the main factor allowing *B. g. tritici* to evade *Pm4* resistance. We also show that SvrPm4, but not its inactive variant svrPm4, is recognized by the NLR Pm1a. Together, these findings represent a substantial step forward in understanding the resistance mechanisms of *Pm4* and open new avenues for studying multi-pathogen resistance provided by KFPs in cereals.

## Results

### Powdery mildew mutants that simultaneously gain virulence on *Pm4a* and *Pm4b* exhibit mutations in a novel type of effector gene

**Fig. 1:**
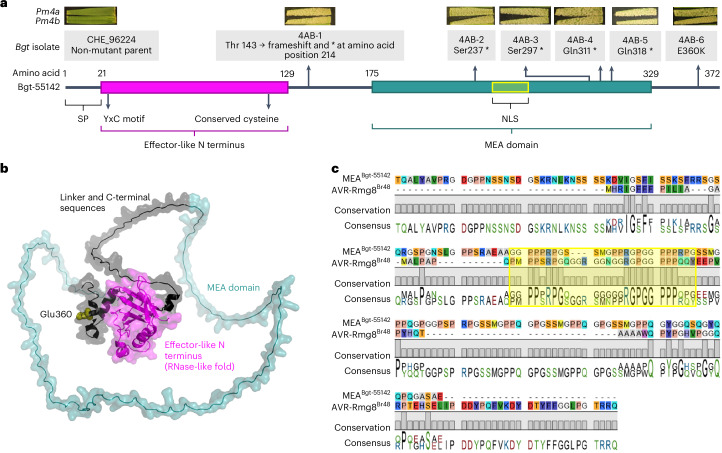
*Pm4* gain-of-virulence mutants all exhibit mutations in *Bgt-55142*, encoding a non-canonical effector. **a**, Bgt-55142 protein structure, indicating the signal peptide (SP), predicted domains (described in detail in Extended Data Fig. 2d) and an NLS predicted by NLStradamus. The phenotypes and genotypes of the *B. g. tritici* gain-of-virulence mutants on *Pm4a* and *Pm4b* are shown above. STOP codons are represented by asterisks, *. **b**, Cartoon and surface representation of the AlphaFold3 structural prediction of Bgt-55142, with domains shown in different colours and labelled accordingly. Glutamate at position 360, altered in mutant 4AB-6 to a lysine, is represented by spheres and indicated in brown. The predicted aligned error is depicted in Extended Data Fig. 2b. **c**, Sequence alignment of the MEA domain of Bgt-55142 (amino acids 175 to 329) and AVR-Rmg8 reveals a region of high identity. The protein variant of the wheat blast isolate Br48 has been used for the alignment. In particular, the RGPGGPPP motif is 100% conserved and overlaps with the NLS (in yellow; Extended Data Fig. 2c).

**Extended Data Fig. 1:**
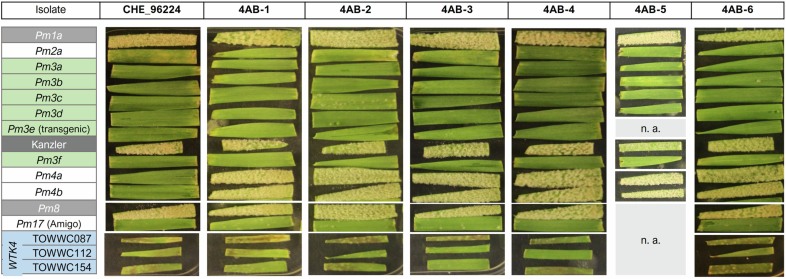
AvrPm4 mutants are virulent on Pm4-containing lines. The *AvrPm4* mutants exhibit specific gain of virulence on *Pm4a* and *Pm4b* near isogenic lines (NILs) but no virulence on various other *R* genes for which their parental isolate CHE_96224 is avirulent. Mutant 4AB-5 died shortly after sequencing and it was not possible to perform phenotyping on all *Pm* lines. Cultivars susceptible to CHE_96224 (the *Pm1a* NIL, the *Pm8* NIL and Kanzler) are highlighted in grey. The NILs containing *Pm3* alleles are depicted in green, and the three *Ae. tauschii* lines containing *WTK4* are in blue. The remaining *Pm* lines are shown in white.

To identify the *B. g. tritici* effector recognized by Pm4, we used the UV-mutagenesis-based approach AvrXpose^22^ and mutagenized the reference *B. g. tritici* isolate CHE_96224, avirulent on near-isogenic wheat lines containing *Pm4a* or *Pm4b*. Six mutants with gain of virulence on both *Pm4* alleles were identified (hereafter, *AvrPm4* mutants). By phenotyping the *AvrPm4* mutants on a set of resistant wheat tester lines, we observed that their gain of virulence was specific to *Pm4* and did not affect avirulence on other *Pm* genes (Extended Data Fig. 1). Whole-genome sequencing revealed that all six *AvrPm4* mutants had mutations in the coding sequence of the effector gene *Bgt-55142* (Fig. 1a). This was the only commonly mutated gene among all six *AvrPm4* mutants (Supplementary Tables 1 and 2), suggesting that *Bgt-55142* is *AvrPm4*. We therefore focused on *Bgt-55142* for further analyses.

**Extended Data Fig. 2:**
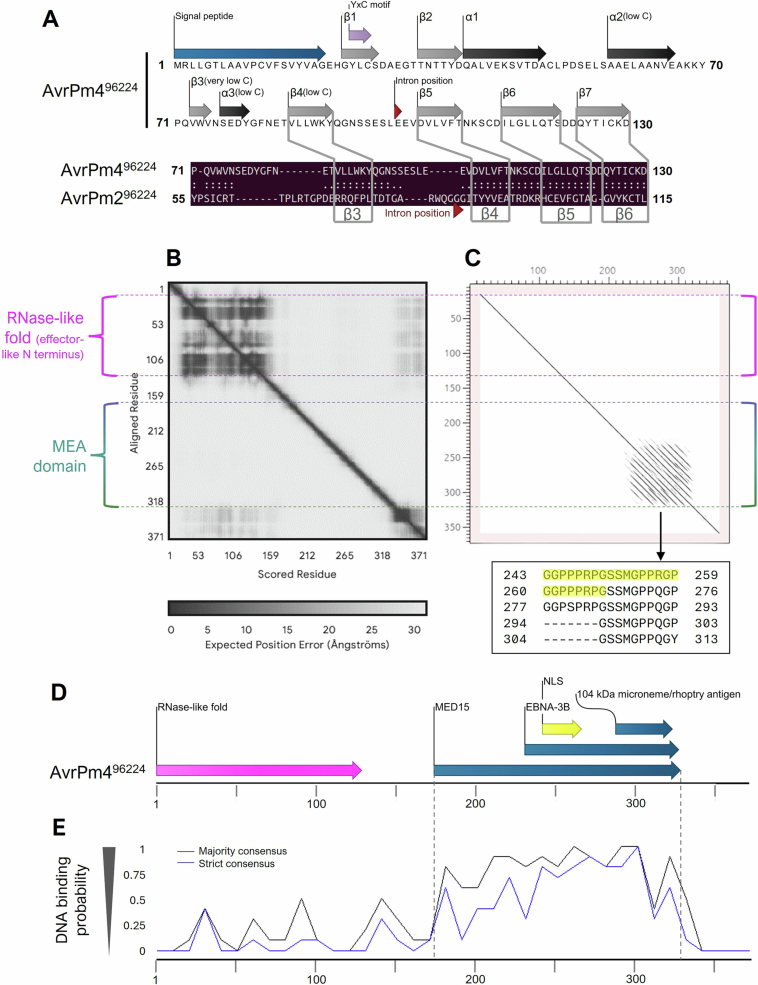
AvrPm4 has an RNase-like N-terminus and an unstructured, highly repetitive, DNA binding MEA domain. (A) Bgt-55142 has an RNase-like N terminus, structurally similar to previously described *B. g. tritici* Avr effectors, such as AvrPm2^72,73^. α-helices and β-strands are depicted on the sequence of Bgt-55142, with indications whether the prediction had a low confidence (low C). TMalign^61^ was used to compare the effector-like part of Bgt-55142 with full length AvrPm2. The TM-alignment of the final part of both Avrs is shown here. In the alignment, a double point indicates high sequence identity and a single point low sequence identity. The intron position in the translated sequence is shown by a red triangle. Its conserved position indicates a common evolutionary origin of the RNase-like fold in both effectors. (**B**) The predicted aligned error (PAE) of the Alphafold prediction (shown in Fig. 1c) highlights a high quality of the prediction (low error) in the effector-like N terminus with an RNase-like fold (in pink), and a poor prediction of the MEA domain (in blue/green). (**C**) A dot plot (visual alignment) of AvrPm4^96224^ against itself at protein level reveals multiple duplicated fragments in the second half of the MEA domain. The duplicated fragments are 17 (or 10) amino acids long, glycine and proline rich, and contain a nuclear localisation signal (NLS) predicted by NLStradamus (in yellow). (**D**) The domain prediction from NCBI conserved domain search reveals three overlapping hits (in blue), with the MED15 domain being the largest (aa 175-329), containing an EBNA-3B (aa 231-328) and a 104 kDa microneme/rhoptry antigen (aa 288-324) domains, with E-values of 4.23e^−3^, 2.83e^−3^ and 1.07e^−3^, respectively. The NLS (aa 243-267) is contained in both the EBNA-3B and the MED15 domain (in yellow). The first 129 amino acids have an RNase-like fold (in pink). (**E**) Density plot of DNA binding probability of AvrPm4 predicted by DP-bind. Binding (value = 1) and non-binding amino acid residues (value = 0) were binned in bins of 10 amino acids, and the average value of the bin is depicted in the plot. The black line indicates the majority consensus (at least 2 of the 3 DP-bind models predict the residue to bind DNA) and the blue line indicates the strict consensus (all 3 models predict the residue to be DNA binding). Raw data from DP-bind are in Supp. Table S6.

Intriguingly, five *AvrPm4* mutants had nonsense mutations in *Bgt-55142* leading to a truncated protein or frameshifts, while only one (4AB-6) had a missense mutation leading to an amino acid change (E360K; Fig. 1a,b). *Bgt-55142* is highly expressed at the haustorial stage^28,29^ and encodes a protein with a signal peptide and an amino-terminal domain with a predicted RNase-like structure, including the characteristic YxC motif and the conserved intron position found in all previously identified *B. g. tritici* Avr proteins (Fig. 1a and Extended Data Fig. 2a)^28–30^. However, it differs from typical powdery mildew Avrs in length and structure, being considerably larger (372 amino acid residues) and containing a carboxy-terminal domain, which is predicted with low confidence by AlphaFold3, probably due to its repetitive and glycine- and proline-rich nature (Fig. 1a–c and Extended Data Fig. 2b,c). On the basis of a conserved domain search via the National Center for Biotechnology Information (NCBI), this domain shows sequence similarity to a MED15 domain, an EBNA-3B domain and a 104-kDa microneme/rhoptry antigen domain (Extended Data Fig. 2d), the first two being previously implicated in transcriptional regulation (see also ‘Discussion’)^31,32^. Given that the regions of sequence similarity with these three domains overlap, we defined the C-terminus of Bgt-55142 as a unique domain, which we renamed MEA (each bold letter representing one of the three identified hits: **M**ED15, **E**BNA-3B and 104-kDa microneme/rhoptry **a**ntigen; Fig. 1a,b and Extended Data Fig. 2d). The MEA domain contains five repeated sequences (three complete, with 17 amino acids, and two incomplete, with 10 amino acids) as well as a predicted nuclear localization signal (NLS) (Extended Data Fig. 2c,d). Furthermore, the software DP-bind indicates a high probability of binding DNA, particularly in the MEA domain of Bgt-55142 (Extended Data Fig. 2e).

The identity of the wheat blast effector AVR-Rmg8, recognized by Pm4 (Rmg8/Rmg7), is known^12–14^. We aligned its protein sequence with Bgt-55142 and found a region with high amino acid identity, corresponding to part of the MEA domain of Bgt-55142, which also contains the predicted NLS. Notably, an eight-amino-acid glycine- and proline-rich motif (RGPGGPPP) is identical between the two effectors (Fig. 1c).

### *Bgt-55142*^*96224*^ expression induces cell death in wheat protoplasts

**Fig. 2:**
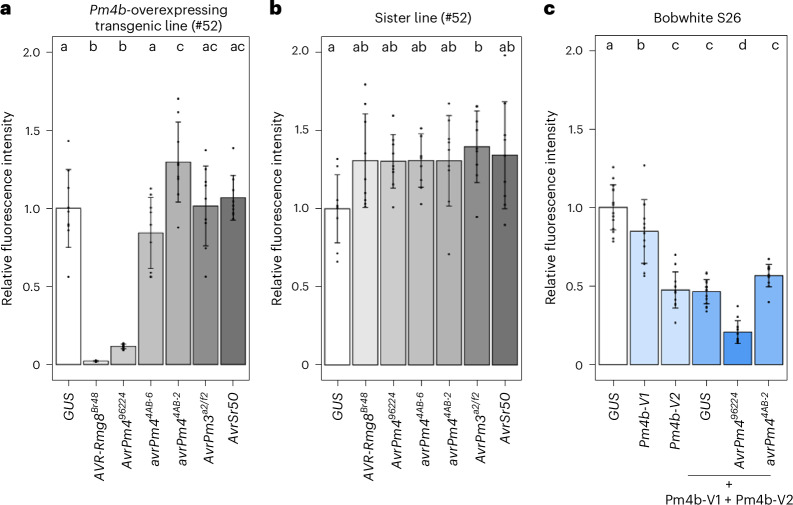
*AvrPm4*^*96224*^ induces cell death in *Pm4b*-containing wheat protoplasts. **a**,**b**, Protoplasts generated from a transgenic line overexpressing *Pm4b* (**a**) and its *Pm4b*-free sister line (**b**) were transfected with different *Avr* effectors. *AVR-Rmg8* was used as a positive control, while *AvrPm3*^*a2/f2*^ and *AvrSr50* served as negative controls. In the presence of *Pm4b*, *AvrPm4*^*96224*^ induced cell death in wheat protoplasts, in contrast to the mutant and truncated versions *Bgt-55142*^*4AB-2*^ and *Bgt-55142*^*4AB-6*^. **c**, Transfection of *Pm4b-V1*, *Pm4b-V2* and *AvrPm4*^*96224*^, but not *Bgt-55142*^*4AB-2*^ (*avrPm4*^*4AB-2*^), induced cell death in protoplasts of the wheat cultivar Bobwhite S26. *Pm4b-V2* shows autoactivity, leading to increased cell death in all tests containing *Pm4b-V2*. For all three panels, each treatment was assessed with two technical and three biological replicates, and the experiments were repeated three times (total *n* = 9 biological replicates per experiment). The height of the bars shows the mean value for all the replicates, and the whiskers show the standard deviation. Statistical significance (*P* < 0.05) was determined using a two-sided analysis of variance (ANOVA) followed by Tukey’s honestly significant difference (HSD) test. Bars with the same letter are not significantly different.

**Extended Data Fig. 3:**
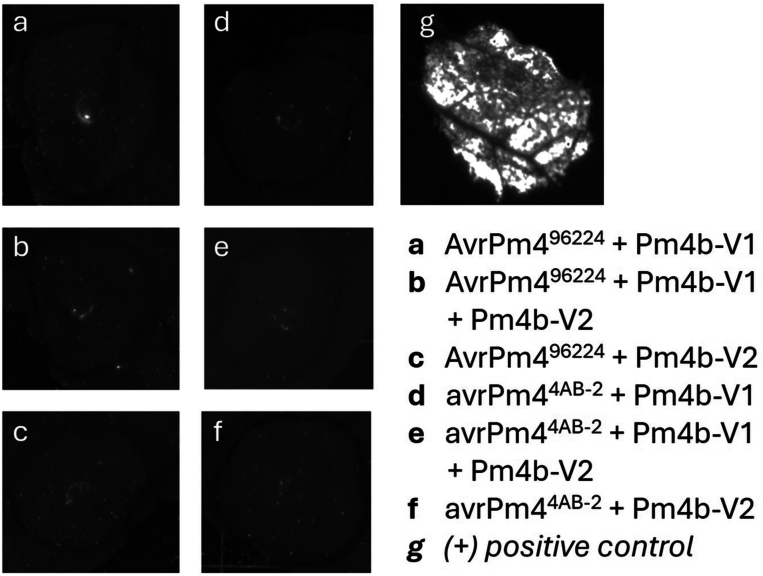
*AvrPm4*^*96224*^ does not induce cell death in co-expression with *Pm4* in *N. benthamiana.* **a**–**g**, The cell death signal was measured 6 days post infiltration. For all treatments, 10% P19 was co-infiltrated. At least 3 replicates per treatment were infiltrated.

To validate *Bgt-55142* as *AvrPm4*, we performed a cell death assay in wheat protoplasts, similar to the one performed for *AVR-Rmg8* (ref. ^12^). Gene sequences of the avirulence form and two mutated forms of *Bgt-55142* (*Bgt-55142*^*96224*^, *Bgt-55142*^*4AB-6*^ and *Bgt-55142*^*4AB-2*^), along with those of known control *Avr*s (*AVR-Rmg8*, *AvrPm3*^*a2/f2*^ and *AvrSr50)*, were codon-optimized for expression in planta and cloned into expression vectors without signal peptides. The constructs were cotransfected with a YFP reporter into protoplasts of the transgenic Bobwhite S26 wheat line stably expressing *Pm4b-V1* and *Pmb4b-V2*, previously described^11^. A non-transgenic sister line lacking *Pm4b* served as a negative control. We observed a strong reduction in relative YFP fluorescence in the *Pm4b*-overexpressing line upon co-expression with *AVR-Rmg8* and *Bgt-55142*^*96224*^, but not in the sister line (Fig. 2a,b). In contrast, neither of the two *Bgt-55142* mutant alleles, *Bgt-55142*^*4AB-6*^ and *Bgt-55142*^*4AB-2*^, showed a reduction in relative YFP fluorescence; nor did the negative controls *Avr-Pm3*^*a2/f2*^ and *Avr-Sr50* (Fig. 2a,b). This indicates that *Bgt-55142*^*96224*^, from now on referred to as *AvrPm4*^*96224*^, is recognized in a *Pm4b*-dependent manner and subsequently triggers cell death. We then transfected *Pm4b-V1* and *Pm4b-V2* in protoplasts of the wheat line Bobwhite S26 (lacking *Pm4*), alone and in combination with each other. *Pm4b-V2* reduced fluorescence intensity by itself and in combination with *Pm4b-V1*, suggesting that it is partially autoactive (Fig. 2c). When transfecting both *Pm4b-V1* and *Pm4b-V2* in combination with *AvrPm4*^*96224*^, we observed a significant reduction in fluorescence intensity compared with *avrPm4*^*4AB-2*^ (Fig. 2c), further confirming *Pm4b*-mediated cell death induction by *AvrPm4*^*96224*^ in wheat. Co-expression of *AvrPm4*^*96224*^ with *Pm4* in *N. benthamiana* did not lead to cell death, indicating that one or more additional genetic components, present in wheat but not in *N. benthamiana*, are needed for triggering *Pm4*-mediated immunity (Extended Data Fig. 3).

### Pm4 directly interacts with AvrPm4, resulting in its localization to the endoplasmic reticulum network

**Fig. 3:**
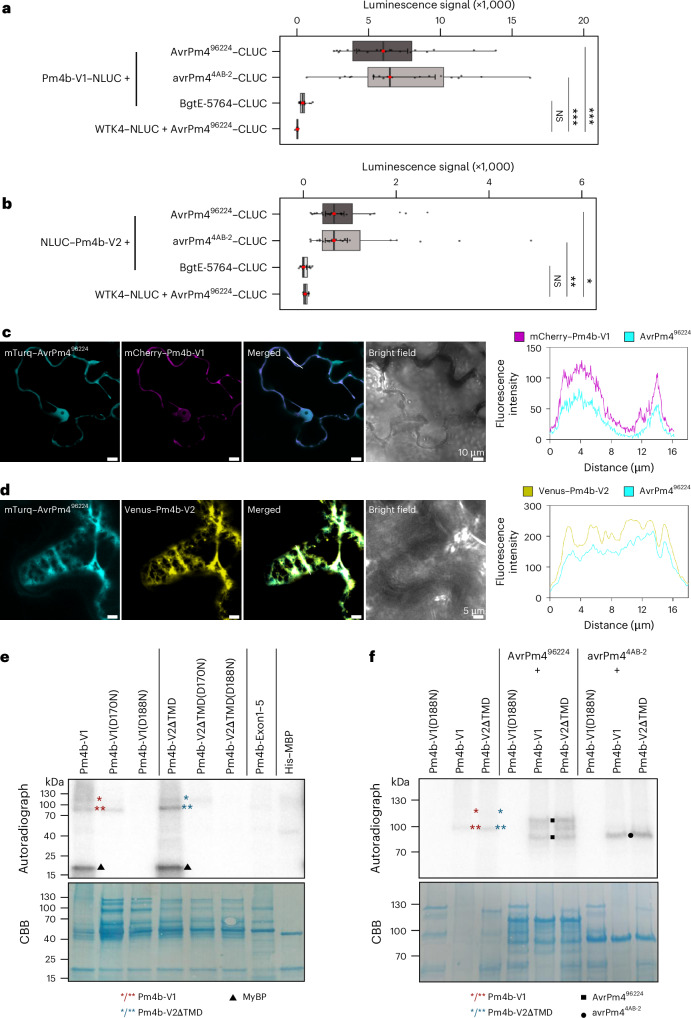
AvrPm4^96224^ interacts with both Pm4b isoforms V1 and V2. **a**,**b**, Split-luciferase assay showing the interaction of NLUC-tagged Pm4b-V1 (**a**) and NLUC-tagged Pm4b-V2 (**b**) with CLUC-tagged AvrPm4^96224^ and CLUC-tagged avrPm4^4AB-2^ in *N. benthamiana*. Co-expression of the two *Pm4b* isoforms with *BgtE-5764* and of *AvrPm4*^*96224*^ with *WTK4* served as negative controls. AvrPm4^96224^ interacts with both Pm4b isoforms. Each treatment was assessed with two technical and six biological replicates, and the experiments were repeated three times (total *n* = 18 biological replicates). In each box plot, the median is shown as a central line in a box that spans from the 25th to the 75th percentile. The inner whiskers show the 95% confidence interval of the median, and the outer whiskers show the most extreme data points within 1.5 times the interquartile range. Significance and *P* values (significance threshold of *P* < 0.05) were calculated with a two-sided ANOVA followed by multiple comparisons of means (contrast analyses) with Bonferroni correction (***P ≤ 0.001; **P ≤ 0.01; *P ≤ 0.05; NS, not significant). **c**,**d**, Subcellular colocalization in *N. benthamiana* of mTurquoise–AvrPm4^96224^ (cyan), Pm4b-V1 (magenta) and Pm4b-V2 (yellow). When co-expressed with Pm4b-V1, AvrPm4^96224^ colocalizes to the cytoplasm (**c**; scale bars, 10 μm), while co-expression with Pm4b-V2 redirects AvrPm4 to the ER (**d**; scale bars, 5 μm). The experiment was repeated twice. **e**, In vitro kinase assay showing autophosphorylation and transphosphorylation activity of purified Pm4 variants in the presence of [ɣ-^32^P]ATP. All constructs shown have an MBP tag. All reactions were supplemented with the common substrate MyBP (21 kDa, indicated by triangles). Constructs included *Pm4b-V1*, *Pm4b-V2ΔTMD* and their kinase-dead mutants (D170N and D188N), as well as a truncated construct containing only the kinase domain (*Pm4b-Exon1–5*; calculated protein size, 77.5 kDa). His-MBP alone served as a negative control. Wild-type Pm4b-V1 (calculated protein size, 106 kDa) and Pm4b-V2ΔTMD (calculated protein size, 107 kDa) were found to migrate at a size of around 120 kDa (see **f** and Extended Data Fig. 5a for better resolution). Wild-type Pm4b-V1 and Pm4b-V2ΔTMD showed kinase activity (indicated by a single asterisk). Truncated proteins migrating at around 90 kDa (indicated by a double asterisk) were also detected. The substitutions D170N and D188N reduced or abolished kinase activity, respectively. Coomassie-stained proteins on polyvinylidene difluoride (PVDF) membranes served as loading controls. CBB, Coomassie brilliant blue. **f**, In vitro phosphorylation assay of AvrPm4^96224^ and avrPm4^4AB-2^ by Pm4. All constructs shown have an MBP tag. Pm4b-V1 and Pm4b-V2ΔTMD were incubated with AvrPm4^96224^ and avrPm4^4AB-2^ in the presence of [ɣ-^32^P]ATP. AvrPm4^96224^ (110 kDa, with truncated products ranging between 70 kDa and 100 kDa (Extended Data Fig. 5b), indicated by two single squares) and avrPm4^4AB-2^ (indicated by a circle) were phosphorylated by both Pm4b isoforms. As a control, the same AvrPm4 constructs were incubated with kinase-dead Pm4b-V1(D188N). The kinase assays were repeated twice. Source data

**Extended Data Fig. 4:**
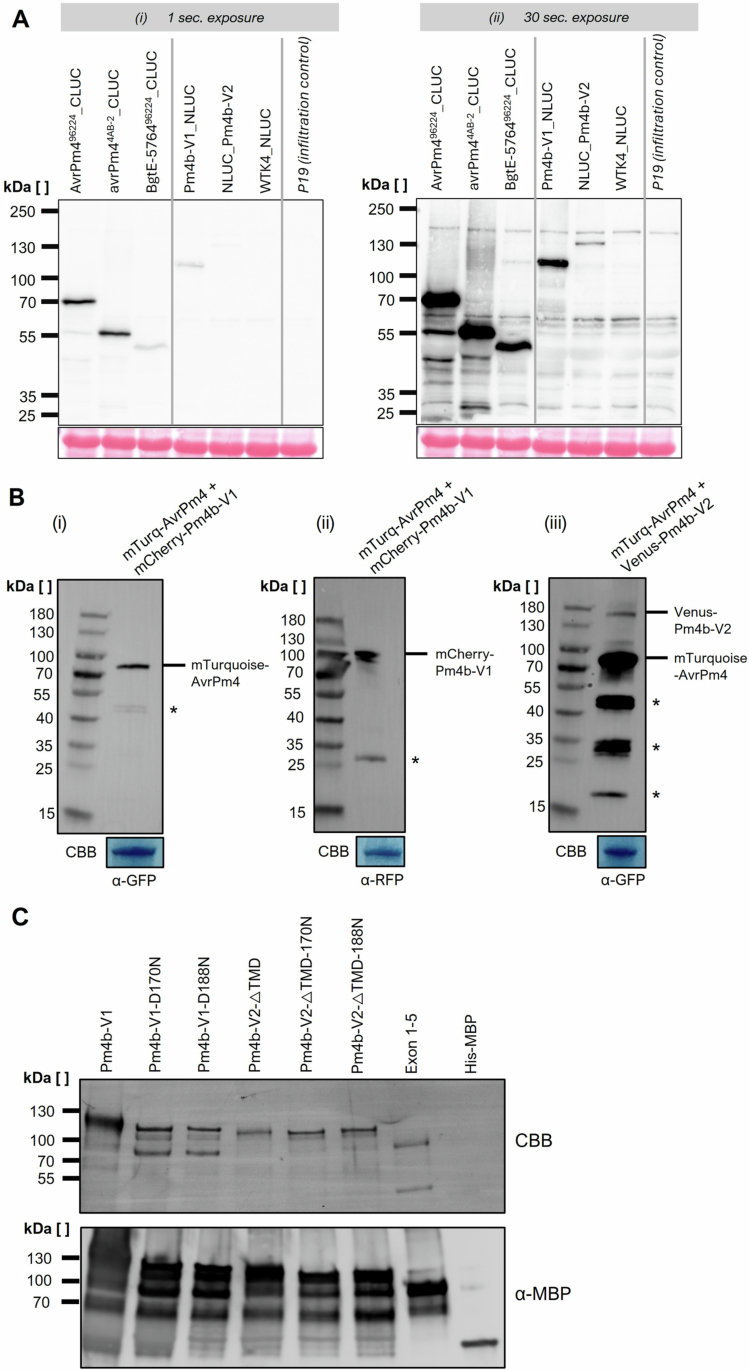
Western blots of different AvrPm4 and Pm4 constructs. (**A**) Protein levels of the different constructs used for the split luciferase experiment (Fig. 3a, b). The same membrane was imaged for 1 s (i) or 30 s (ii) exposure time. Ponceau staining below the blots shows equal protein loading. (**B**) Detection of full-length mTurquoise-AvrPm4 (65 kDa) co-expressed with either mCherry-Pm4b-V1 (90 kDa) or Venus-Pm4b-V2 (113 kDa) in the *N. benthamiana* samples used for the co-localisation experiments. The unlabelled bands observed in the WB (indicated with an asterisk) correspond to partial degradation products of the target protein and/or free fluorophore. (**C**) Coomassie Brilliant Blue G-250 staining and western blot of purified MBP-tagged proteins used in the in-vitro kinase assay. All immunoblotting experiments have been performed twice with similar results. Source data

Multiple tandem kinases interact with their corresponding Avr effectors as part of their resistance function^7,8,17^. We therefore investigated whether Pm4 binds AvrPm4 in a split-luciferase assay in *N. benthamiana*. We found that both AvrPm4^96224^ and the truncated avrPm4^4AB-2^ interacted with both Pm4b isoforms, Pm4b-V1 and Pm4b-V2 (Fig. 3a,b). In contrast, we did not observe an interaction between Pm4 and the unrelated effector BgtE-5764 or between AvrPm4^96224^ and WTK4, another KFP, confirming the specificity of the AvrPm4–Pm4 interaction (Fig. 3a,b). We consistently observed a stronger interaction signal between AvrPm4 and Pm4b-V1 than between AvrPm4 and Pm4b-V2, possibly reflecting differential protein accumulation between the isoforms (Extended Data Fig. 4a). The fact that the virulence variant avrPm4^4AB-2^ also interacts with Pm4 suggests that the interaction alone is not sufficient to trigger an immune response.

As previously reported, Pm4b-V1 predominantly localizes to the cytoplasm in the absence of Pm4b-V2, whereas Pm4b-V2 or Pm4b-V1–Pm4b-V2 complexes localize to the endoplasmic reticulum (ER)^11^. To explore whether AvrPm4 and Pm4b share similar localization patterns indicative of a functional interaction, we assessed their subcellular localization in *N. benthamiana*. We co-expressed mTurquoise–AvrPm4^96224^ with either mCherry–Pm4b-V1 or Venus–Pm4b-V2 and examined their subcellular localization via confocal microscopy. Intensity plot analyses revealed that mTurquoise–AvrPm4^96224^ colocalizes with mCherry–Pm4b-V1 in the cytoplasm (Fig. 3c and Extended Data Fig. 4b), whereas co-expression with Venus–Pm4b-V2 relocalizes AvrPm4^96224^ to the ER network (Fig. 3d and Extended Data Fig. 4b). These findings provide additional evidence supporting the interaction between the two Pm4b isoforms and AvrPm4^96224^.

### Pm4 exhibits kinase activity and phosphorylates AvrPm4

Earlier work has shown that both Pm4 isoforms share a kinase domain that exhibits sequence characteristics of an active kinase. Furthermore, multiple loss-of-function mutants affecting this domain confirmed its essential role in resistance^11^. We therefore tested whether Pm4 exhibits kinase activity and whether AvrPm4 is a substrate for phosphorylation by Pm4.

**Extended Data Fig. 5:**
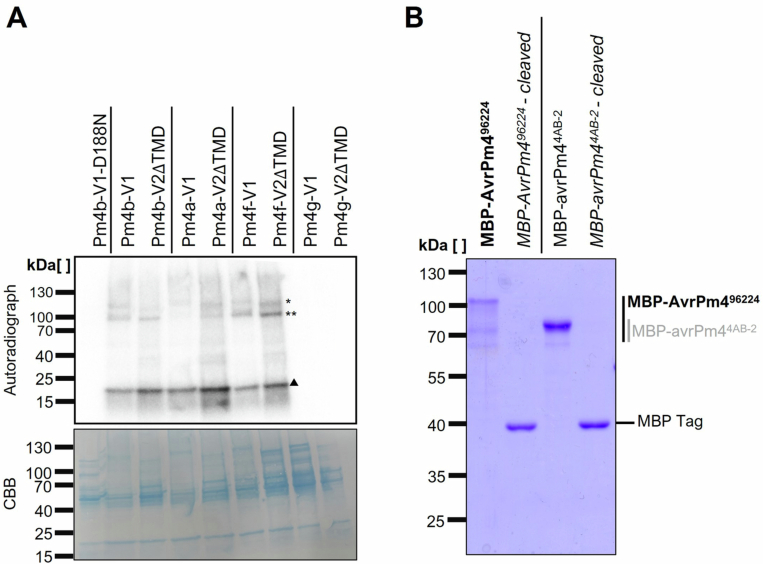
Different Pm4 variants show different kinase activity and AvrPm4 precipitates without MBP tag. (**A**) *In vitro* autophosphorylation and transphosphorylation assays in the presence of [ɣ-32P] and myelin basic protein using purified Pm4-V1 and V2∆TMD of the *Pm4* alleles: P*m4a, Pm4b, Pm4f*, and *Pm4g*. All constructs shown have an MBP tag. Different Pm4 allelic variants consistently migrate at around 120 kDa, and a smaller product at around 90 kDa (indicated by asterisks). Coomassie Brilliant Blue R-250 stained PVDF membrane is indicated as a loading control. This assay has been performed twice. (**B**) MBP tagged AvrPm4 was purified as described in material and methods. An aliquot of the purified sample was incubated with enterokinase to cleave off the MBP tag. The four samples were subsequently run on an SDS-PAGE and Coomassie stained. MBP-AvrPm4^96224^ (predicted size 80 kDa) migrates at 110 kDa, with truncated versions between 100 kDa and 70 kDa. MBP-avrPm4^4AB-2^ (67 kDa) migrates between the 70 kDa and 85 kDa mark and shows a truncated occurring version at 70 kDa. After cleavage with enterokinase, the MBP tag is visible at 42 kDa. AvrPm4 is no longer detectable on the gel, likely due to its insolubility in absence of the MBP tag, resulting in precipitation. Source data

We established an in vitro assay by expressing full-length Pm4b-V1 (kinase domain + C2C domain) and a truncated, soluble version of Pm4b-V2 (Pm4b-V2ΔTMD, kinase domain + C2D domain, lacking the transmembrane and PRT-C domains) with a maltose binding protein (MBP) tag in *Escherichia coli*. Both isoforms were purified using amylose resin, and protein integrity was assessed via Coomassie staining and immunoblotting (Fig. 3e,f and Extended Data Figs. 4c and 5). Pm4b-V1 (calculated protein size, 106 kDa) and Pm4b-V2ΔTMD (calculated protein size, 107 kDa) purification showed stronger protein accumulation at around 120 kDa and some smaller products, potentially truncated Pm4 proteins (Fig. 3e and Extended Data Fig. 4c).

Both Pm4b-V1 and Pm4b-V2ΔTMD exhibited in vitro kinase activity and autophosphorylation (Fig. 3e, asterisks). Two bands were visible on the autoradiographs, corresponding to the previously mentioned larger band at around 120 kDa and a smaller band at around 90 kDa (Fig. 3e,f and Extended Data Fig. 5, asterisks). This indicates that either the smaller form is catalytically active, or a phosphorylation target of the larger protein is present in the extract. Importantly, both Pm4b isoforms also exhibited transphosphorylation activity, phosphorylating myelin basic protein (MyBP) as an exogenous substrate (Fig. 3e, triangle). A truncated construct containing only the kinase domain (exons 1–5, lacking a C2 domain) did not exhibit kinase activity, suggesting that the C2C domain in Pm4b-V1 and the C2D domain in Pm4b-V2 are required for kinase activity (Fig. 3e).

We previously identified two EMS mutants compromised in *Pm4* resistance to powdery mildew, each carrying a non-synonymous mutation in the sequence encoding a conserved motif of the kinase domain: a D170N substitution in the HLDLKPAN motif of the catalytic loop and a D188N substitution in the activation loop^11^. To assess the functional impact of these substitutions, we introduced each substitution into both Pm4b isoforms and tested auto- and transphosphorylation activity using MyBP as a substrate. Auto- and transphosphorylation were strongly reduced by D170N and completely abolished by the D188N substitution (Fig. 3e), demonstrating a correlation between Pm4 kinase activity and resistance.

We further hypothesized that kinase activity may distinguish functional from non-functional *Pm4* allelic variants. To test this, we evaluated the in vitro kinase activity of the proteins encoded by alleles *Pm4a*, *Pm4f* and *Pm4g*. *Pm4a* has been described to have a largely overlapping resistance spectrum against *B. g. tritici* with *Pm4b*, while *Pm4f* provides wheat blast resistance by recognizing the wheat blast effector *AVR-Rmg8*. Importantly, *Pm4g* was reported to lack resistance activity against both pathogens and is thus considered a non-functional allele^11–13^. We observed that both Pm4a and Pm4f isoforms exhibited auto- and transphosphorylation activity comparable to that of Pm4b, whereas Pm4g did not show detectable kinase activity (Extended Data Fig. 5a), further supporting the hypothesis that kinase activity is crucial for Pm4 resistance function.

Given the importance of kinase activity for *Pm4* resistance and cell death induction, along with its ability to transphosphorylate proteins such as MyBP and its interaction and colocalization with AvrPm4, we investigated whether Pm4 can directly phosphorylate AvrPm4. To this end, we expressed MBP-tagged AvrPm4^96224^ and the truncated virulence variant MBP–avrPm4^4AB-2^ in *E. coli*, followed by protein purification using amylose resin. We incubated the purified effectors with Pm4b-V1, Pm4b-V2ΔTMD and the kinase-inactive mutant Pm4b-V1(D188N) as a negative control. Despite similar size ranges to Pm4b-V1 and Pm4b-V2ΔTMD (indicated by asterisks), MBP–AvrPm4^96224^ and MBP–avrPm4^4AB-2^ (indicated by two squares and a circle, respectively) were clearly distinguishable from Pm4-derived bands (Fig. 3f and Extended Data Fig. 5b). Importantly, we observed phosphorylation of AvrPm4^96224^ in the presence of Pm4b-V1 and Pm4b-V2ΔTMD but not in the presence of the kinase-dead mutant Pm4b-V1(D188N) (Fig. 3f, square). Surprisingly, despite its inability to trigger cell death (Fig. 2), the virulence variant avrPm4^4AB-2^ was also phosphorylated by both Pm4 isoforms (Fig. 3f, circle). These results confirm AvrPm4–Pm4 interaction in vitro but also suggest that phosphorylation of the Avr alone is insufficient to trigger an immune response.

### *B. g. tritici* virulence on *Pm4* is controlled by a suppressor locus on chromosome 8

Loss-of-function UV mutants of *AvrPm4* result in virulence on both *Pm4a* and *Pm4b* (Fig. 1a). To elucidate the natural mechanism of *B. g. tritici* race specificity on *Pm4a* and *Pm4b*, we phenotyped a set of 78 *B. g. tritici* isolates selected from a worldwide collection^33^ on the near-isogenic lines (NILs) W804/8*Fed (*Pm4b*) and Khapli/8*CC (*Pm4a*) and their corresponding susceptible controls Federation (Fed) and Chancellor (CC). For most isolates, we observed identical virulence phenotypes on both *Pm4* alleles (Supplementary Table 3), consistent with previous findings indicating that *Pm4b* and *Pm4a* have largely overlapping—although not identical—recognition spectra^11^.

**Extended Data Fig. 6:**
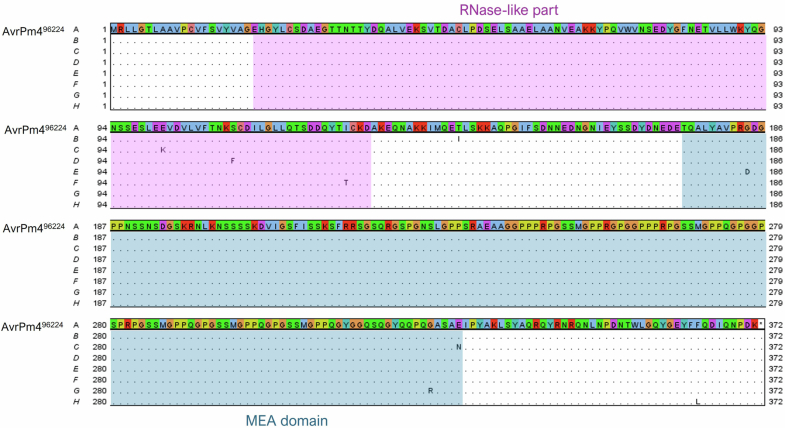
AvrPm4 occurs in eight different protein variants in a selection of 78 *B. g. tritici* isolates. Dots represent non-mutated residues. The RNase-like part and the MEA domain are highlighted with a magenta and a blue box, respectively. The corresponding phenotypes of the protein variants are indicated in Supp. Table S3.

**Extended Data Fig. 7:**
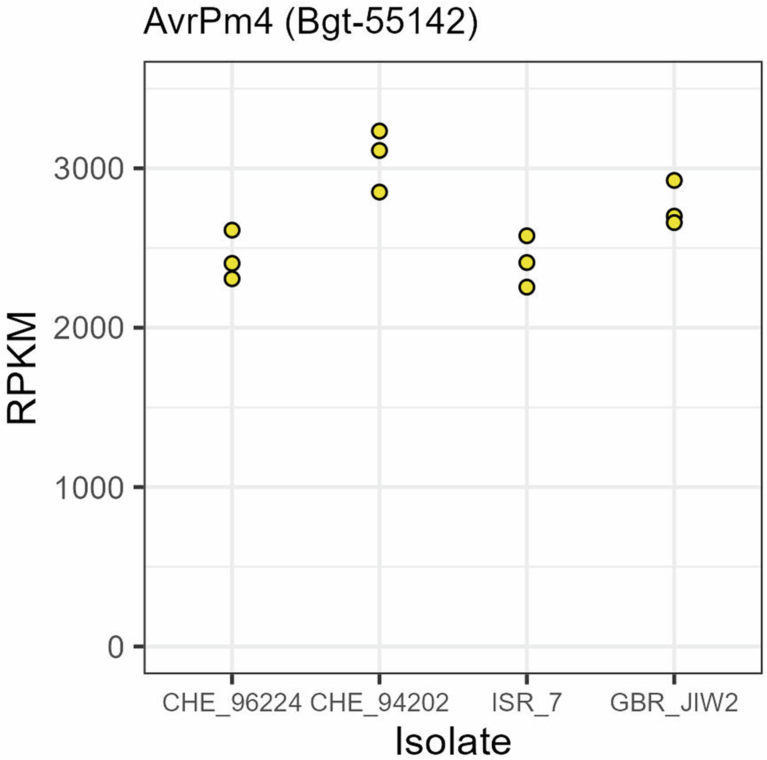
*AvrPm4* (*Bgt-55142*) expression levels in four *B. g. tritici* reference isolates CHE_96224, CHE_94202, ISR_7 and GBR_JIW2. Expression levels are shown as RPKM values at 48 h post infection on the susceptible wheat cultivar ‘Chinese Spring’. Values of three biological replicates are shown as yellow circles.

**Extended Data Fig. 8:**
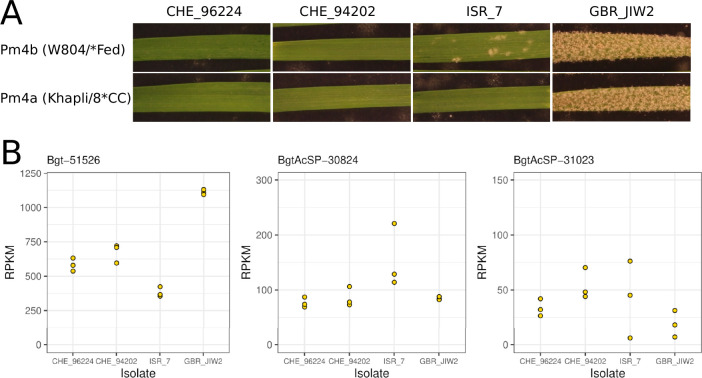
*Pm4b* and *Pm4a* phenotypes and *SvrPm4* candidate gene expression levels in four *B. g. tritici* reference isolates. (**A**) Virulence phenotypes of reference isolates CHE_96224, CHE_94202, ISR_7 and GBR_JIW2 on the near isogenic lines ‘W804/8*Fed’ (*Pm4b*) and ‘Khapli/8*CC’ (*Pm4a*). Pictures were taken eight days post inoculation. (**B**) Expression levels of *SvrPm4* candidate genes *Bgt-51526, BgtAcSP-30824* and *BgtAcSP-31023* in the four *B. g. tritici* reference isolates CHE_96224, CHE_94202, ISR_7 and GBR_JIW2. Expression levels are shown as RPKM values at 48 h post infection on the susceptible wheat cultivar ‘Chinese Spring’. Values of three biological replicates are shown as yellow circles.

We next assessed the genetic diversity of *AvrPm4* in the same subset of 78 *B. g. tritici* isolates. *AvrPm4* was present in all tested isolates, and it was largely conserved, with only 19 out of 78 isolates exhibiting single nucleotide polymorphisms (SNPs) in the coding sequence compared with the reference isolate CHE_96224 (Supplementary Table 3 and Extended Data Fig. 6). Importantly, no correlation was found between the *AvrPm4* genotype and virulence phenotypes on *Pm4b* and *Pm4a* NILs. To rule out expression polymorphisms, we analysed available RNA-sequencing datasets and observed no significant variation in *AvrPm4* expression between *B. g. tritici* isolates with contrasting phenotypes on *Pm4*, such as CHE_96224, CHE_94202 and ISR_7 (all avirulent on *Pm4b*/*Pm4a*) versus GBR_JIW2 (virulent on *Pm4b*/*Pm4a*) (Extended Data Figs. 7 and 8a)^21,29^. Taken together, these observations suggest that additional genetic components beyond *AvrPm4* control virulence on *Pm4*. To identify such additional components, we used two independent approaches: GWAS and biparental QTL mapping.

**Fig. 4:**
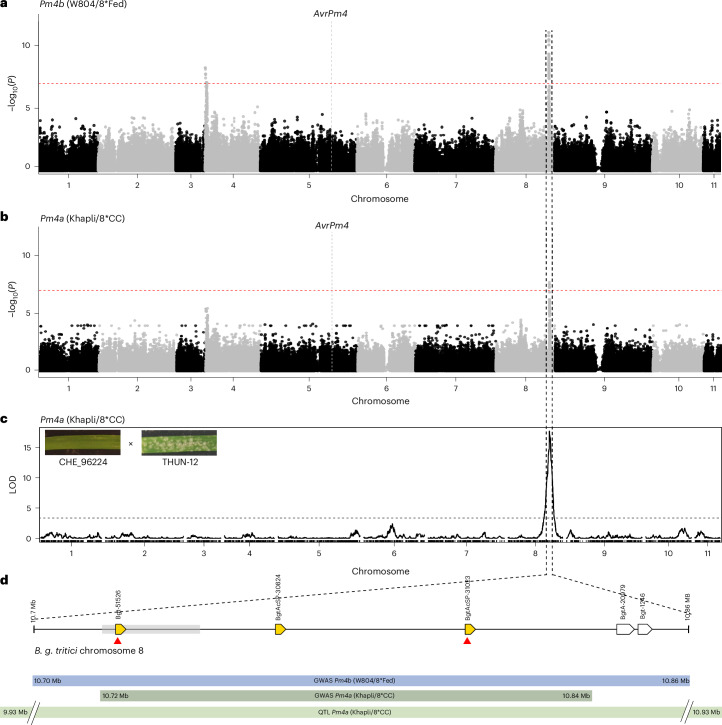
GWAS and QTL mapping identify a common locus on *B. g. tritici* chromosome 8 controlling virulence on *Pm4b* and *Pm4a.* **a**,**b**, GWAS analysis using 473,887 SNPs across the 11 *B. g. tritici* chromosomes, based on phenotypes of 78 isolates of the *Pm4b* NIL W804/8*Fed (**a**) and the *Pm4a* NIL Khapli/8*CC (**b**). The red dashed line indicates the significance threshold at *P* < 0.05 after Bonferroni correction. The genomic position of *AvrPm4* is indicated by a grey dashed line. **c**, Results of a QTL analysis on *Pm4a* NIL Khapli/8*CC, using 118 F_1_ progenies of the biparental mapping population CHE_96224 (avirulent) × THUN-12 (partially virulent). The virulence phenotypes of the parental isolates on the *Pm4a* NIL are shown in the top left corner. The dashed line indicates the significance threshold logarithm of the odds (LOD) value at *P* < 0.05, determined by 1,000 permutations. **d**, Depiction of the genomic locus on chromosome 8 identified via GWAS and QTL analysis. Locus borders were defined on the basis of the GWAS analysis on the *Pm4b* NIL (W804/8*Fed). Candidate secreted effector genes are represented by yellow arrows, and non-effector genes by white arrows. Red triangles indicate candidate effectors exhibiting non-synonymous sequence polymorphisms between *Pm4b*/*Pm4a* virulent and avirulent reference isolates. The grey box indicates the location of the 13 best associated SNPs with the phenotype on *Pm4b* (W804/8*Fed) with an identical *P* value of 8.660015 × 10^−12^. Gene names are based on the gene annotation published for *B. g. tritici* reference isolate CHE_96224 (ref. ^34^). Gene models are not drawn to scale. Genomic confidence intervals identified by GWAS on *Pm4b* (blue), GWAS on *Pm4a* (dark green) or QTL mapping on *Pm4a* (light green) are indicated by coloured bars.

The above-mentioned *B. g. tritici* collection of 78 isolates, phenotyped on the NILs W804/8*Fed (*Pm4b*) and Khapli/8*CC (*Pm4a*), provided the basis for GWAS. Using an avirulent isolate as a reference (CHE_96224), GWAS revealed two significant genetic associations for virulence on *Pm4b*: a strong association at the end of chromosome 8, spanning from 10704663 to 10864069 bp in the reference assembly of CHE_96224, and a less pronounced association at the beginning of chromosome 4 (2736 to 457765 bp) (Fig. 4a). GWAS analysis on *Pm4a* revealed a single significant genetic association located on chromosome 8 (10721247 to 10841388 bp), overlapping with the locus identified for *Pm4b* (Fig. 4b).

To complement the GWAS approach, we performed QTL mapping on *Pm4a*, using a biparental cross (originally described in ref. ^34^) between the *Pm4a/b*-avirulent *B. g. tritici* isolate CHE_96224 and the *B. g. triticale* isolate THUN-12, which displays partial virulence on *Pm4a* but is avirulent on *Pm4b* (Fig. 4c). To do so, we phenotyped 118 F_1_ progenies on the *Pm4a* NIL Khapli/8*CC and conducted a QTL analysis using 119,023 genetic markers between the parental isolates. Remarkably, a single QTL was identified on chromosome 8, spanning from positions 9931004 to 10932716 in the CHE_96224 reference assembly, overlapping with the chromosome 8 locus detected in the GWAS analysis (Fig. 4c).

Consistent with our previous observation that genetic diversity within *AvrPm4* does not correlate with virulence phenotypes on *Pm4b* or *Pm4a*, neither GWAS nor QTL mapping identified a significant genetic association with the *AvrPm4* gene located on chromosome 5 (Fig. 4a–c). Instead, both complementary approaches provide strong evidence for the presence of a major genetic component on chromosome 8 controlling *B. g. tritici* virulence on the *Pm4b* and *Pm4a* resistance alleles. Given that loss-of-function mutations in *AvrPm4* alone are sufficient to result in virulence on *Pm4b* and *Pm4a* (Fig. 1a), it is unlikely that the locus on chromosome 8 contains an additional *Avr* component. We therefore hypothesized that this region contains a suppressor of avirulence (*SvrPm4*), which interferes with AvrPm4 recognition by Pm4.

### *SvrPm4* (*Bgt-51526*^*JIW2*^) suppresses *AvrPm4*/*Pm4*-induced cell death in wheat protoplasts

**Fig. 5:**
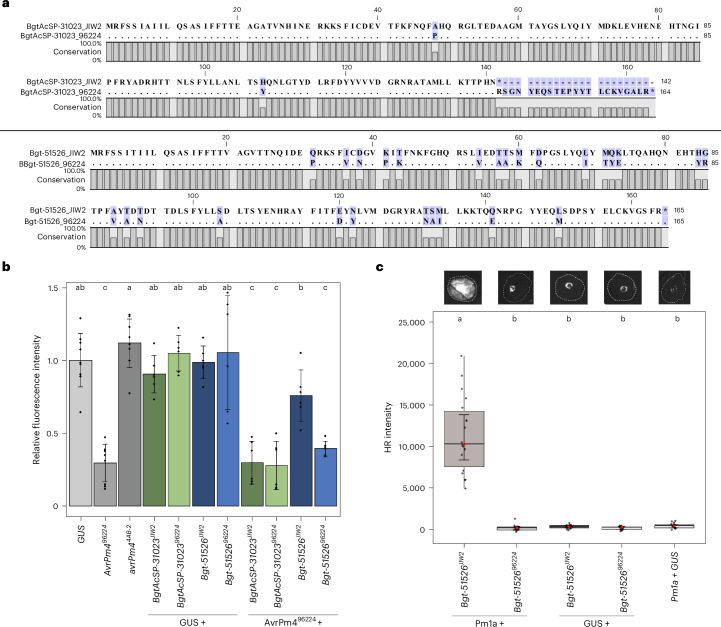
*AvrPm4*^*96224*^-induced cell death in *Pm4*-containing wheat protoplasts is suppressed by co-expression of *SvrPm4*^*JIW2*^. **a**, Protein sequence alignment of SvrPm4 candidates BgtAcSP-31023 and Bgt-51526 haplovariants found in the *Pm4*-virulent isolate GBR_JIW2 and in the *Pm4*-avirulent isolate CHE_96224. GBR_JIW2 is shown as a reference, with polymorphic residues in CHE_96224 highlighted. **b**, Wheat protoplast assay showing the suppression of *AvrPm4*-induced cell death by *SvrPm4*^*JIW2*^. Protoplasts isolated from the transgenic wheat line #52, overexpressing *Pm4b* (used in Fig. 2), were transfected with each *Svr* candidate individually or cotransfected with *AvrPm4*. Presumed suppressor-active candidates (BgtAcSP-31023^JIW2^ and Bgt-51526^JIW2^) and suppressor-inactive candidates (BgtAcSP-31023^96224^ and Bgt-51526^96224^) were tested. The experiment was performed twice with three biological replicates each (*n* = 6 total). The height of the bars shows the mean value for all the replicates, and the whiskers show the standard deviation. **c**, HR response in *N. benthamiana* upon *Agrobacterium*-mediated co-expression of Bgt-51526^JIW2^ or Bgt-51526^96224^ and *Pm1a-HA* or a negative GUS control. Close-up images of the HR response are shown at the top. The full-size image of the corresponding *N. benthamiana* leaf is provided in Extended Data Fig. 10. The assay was performed three times with *n* = 6 leaves per experiment (*n* = 18 total). In each box plot, the median is shown as a central line within a box that spans from the 25th to the 75th percentile. The inner whiskers show the 95% confidence interval of the median, and the outer whiskers show the most extreme data points within 1.5 times the interquartile range. For all experiments, significance and *P* values (significance threshold of *P* < 0.05) were calculated with a two-sided ANOVA followed by Tukey’s HSD test (Supplementary Table 3). Bars or boxes with the same letter are not significantly different.

We defined *SvrPm4* candidate genes within the identified locus on chromosome 8 of the *Pm4*-avirulent isolate CHE_96224 on the basis of the GWAS analysis on *Pm4b* (Fig. 4d). Focusing on effector genes with a predicted signal peptide, we identified three candidate genes within this interval: *Bgt-51526*, *BgtAcSP-30824* and *BgtAcSP-31023*. Analysis of their expression levels during infection in four RNA-sequenced *B. g. tritici* reference isolates exhibiting differential phenotypes on *Pm4b* and *Pm4a* (avirulent: CHE_96224, CHE_94202 and ISR_7; virulent: GBR_JIW2) revealed no major expression polymorphisms (Extended Data Fig. 8a,b). We therefore focused on sequence polymorphisms between avirulent and virulent reference isolates to define promising *SvrPm4* candidates. Whereas *BgtAcSP-30824* did not exhibit any sequence polymorphisms, both *Bgt-51526* and *BgtAcSP-31023* were consistently polymorphic between the *Pm4*-virulent reference isolate GBR_JIW2 and the avirulent isolates CHE_96224, CHE_94202 and ISR_7. We found that the effector protein Bgt-51526^JIW2^ differs by 27 amino acids from Bgt-51526^96224^, whereas BgtAcSP-31023^JIW2^ exhibits two amino acid polymorphisms and a premature stop codon at position 142 of the effector protein, compared with BgtAcSP-31023^96224^, found in avirulent isolates (Fig. 5a). We therefore considered both genes as *SvrPm4* candidates and proceeded to test their ability to suppress *Pm4*-mediated cell death in wheat protoplasts.

We codon-optimized the two candidate genes without signal peptides and cotransfected them with a YFP reporter into protoplasts from the transgenic wheat line #52 stably expressing *Pm4b-V1* and *Pmb4b-V2*, as described above. In the absence of *AvrPm4*, none of the *SvrPm4* candidates triggered cell death in the *Pm4* transgenic line, consistent with our initial hypothesis that the locus on chromosome 8 does not contain an *Avr* but rather an *Svr* factor (Fig. 5b). However, co-expression of *Bgt-51526*^*JIW2*^ with *AvrPm4* significantly reduced the cell death response, whereas neither *Bgt-51526*^*96224*^ nor either of the two *BgtAcSP-31023* variants had any detectable suppressive effect (Fig. 5b). We therefore concluded that *Bgt-51526*^*JIW2*^ (hereafter *SvrPm4*^*JIW2*^) is indeed a suppressor of *Pm4*-mediated cell death, whereas *Bgt-51526*^*96224*^ (hereafter *svrPm4*^*96224*^) is unable to suppress *Pm4* activity.

**Extended Data Fig. 9:**
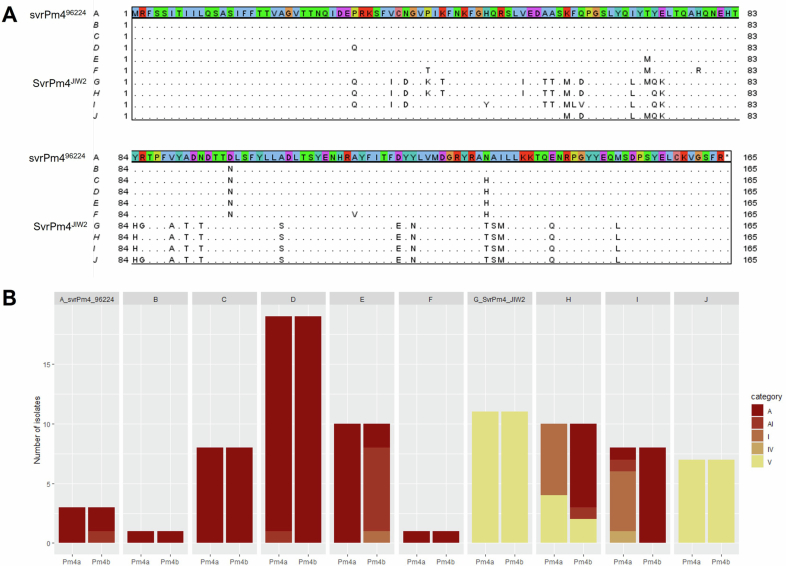
Bgt-51526 (SvrPm4) haplovariants and correlation with virulence phenotypes on *Pm4b* and *Pm4a* near-isogenic lines. (**A**) Multiple sequence alignment of Bgt-51526 haplovariants found in the 78 tested *B. g. tritici* isolates. Bgt-51526 protein sequence of the isolate CHE_96224 (=svrPm4^96224^) is shown as a reference. Polymorphic residues in haplovariants B-J are highlighted. (**B**) Phenotypic spectrum of isolates with the indicated haplovariants on *Pm4a* and *Pm4b* NILs. Phenotypes were assessed 8 days post inoculation on at least three biological replicates and categorized based on observed mildew leaf coverage (avirulent (A) = 0%; avirulent-intermediate (AI) = ( ~ 25%); intermediate (I) = ~ 50%; intermediate-virulent (IV) = ~ 75%; virulent (V) = 100%).

Among the 78 tested *B. g. tritici* isolates, we found extensive sequence variation within the *SvrPm4* gene, identifying ten highly polymorphic haplovariants (Extended Data Fig. 9a and Supplementary Table 3). Importantly, all isolates carrying the inactive variant svrPm4^96224^ exhibit an avirulent phenotype on *Pm4b* and *Pm4a*, whereas all isolates carrying SvrPm4^JIW2^ are virulent on both *Pm4* alleles (Extended Data Fig. 9b), highlighting the importance of SvrPm4 in overcoming *Pm4* resistance. We identified multiple other haplovariants that were exclusively present in either avirulent or virulent isolates, thereby probably representing additional inactive and active SvrPm4 variants, respectively (Extended Data Fig. 9b). Intriguingly, we also found three haplovariants (variants E, H and I in Supplementary Table 3) associated with *B. g. tritici* isolates showing differential phenotypes on *Pm4b* and *Pm4a*, suggesting that *SvrPm4* allelic variation may contribute to the subtle differences in *Pm4b* and *Pm4a* recognition spectra (Extended Data Fig. 9b). Taken together, our data suggest a genetic two-component system controlling virulence on *Pm4* in natural *B. g. tritici* isolates, in which the ability to overcome *Pm4*-mediated resistance is independent of the *AvrPm4* genotype but rather controlled by the presence of the suppressor *SvrPm4*.

### The active suppressor SvrPm4^JIW2^ but not svrPm4^96224^ is recognized by the wheat NLR Pm1a

**Extended Data Fig. 10:**
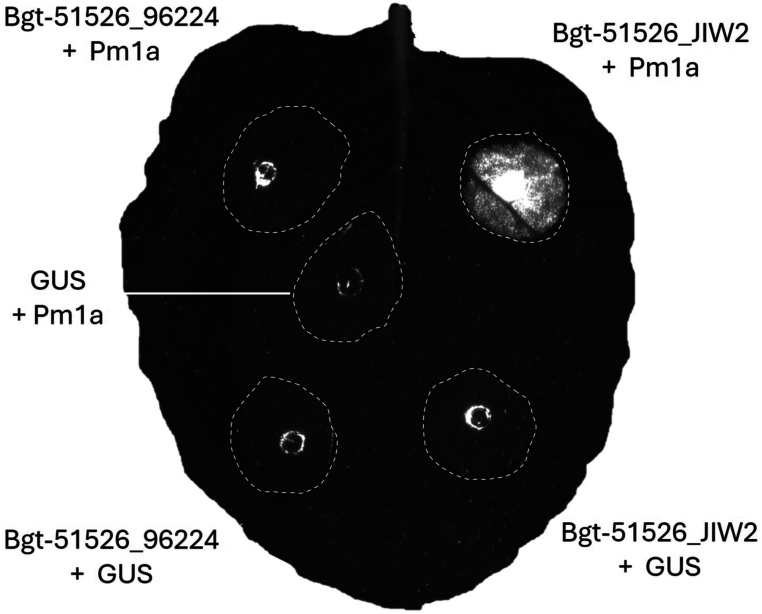
Full-size image of infiltration spots depicted as zoom-ins in Fig. 5c, showing that SvrPm4^JIW2^ but not svrPm4^96224^ is recognised by Pm1a. *Agrobacterium*-mediated co-expression of *Bgt-51526_JIW2* (*SvrPm4*^*JIW2*^) or *Bgt-51526_96224* (*svrPm4*^*96224*^) with Pm1a-HA or a GUS negative control in *N. benthamiana*.

The gene *Bgt-51526* (*SvrPm4*) encodes an RNase-like effector protein that was previously identified as an Avr recognized by the NLR immune receptor encoded by the wheat resistance gene *Pm1a*^35^. To determine whether *SvrPm4*^*JIW2*^ (defined as Bgt-51526_2 variant in ref. ^35^) and the previously undescribed *svrPm4*^*96224*^ variant induce differential HR responses, we co-expressed each variant with *Pm1a* in *N. benthamiana*. Consistent with previous findings, we observed a strong HR response upon co-expression of *SvrPm4*^*JIW2*^ and *Pm1a*, confirming this variant’s avirulence towards *Pm1a*. Note that co-expression of *svrPm4*^*96224*^ and *Pm1a* did not result in an HR response (Fig. 5c and Extended Data Fig. 10). We thus conclude that *SvrPm4*^*JIW2*^ is simultaneously an active suppressor of *Pm4* and an *Avr* of *Pm1a*, whereas the inactive suppressor *svrPm4*^*96224*^ escapes *Pm1a* recognition.

## Discussion

Avirulence on *Pm4* is controlled by at least two genes in *B. g. tritici*. *AvrPm4* is an avirulence factor, and its disruption results in virulence on both *Pm4a* and *Pm4b*. Furthermore, *AvrPm4*-triggered immunity is suppressed by *SvrPm4*. This multi-component system was uncovered using UV mutagenesis, GWAS and QTL mapping, illustrating how the combination of gene identification tools allows the dissection of genetically complex systems in biotrophic fungal plant pathogens such as *B. g. tritici*.

Previously identified Avr effectors in *B. g. tritici* encode small (100–155 amino acid residues) RNase-like effectors recognized by NLR receptors^23^. In contrast, AvrPm4, recognized by the KFP Pm4, is a 372-residue chimeric protein with an N-terminal RNase-like domain and a C-terminal MEA domain and differs substantially from previously identified *B. g. tritici* Avr proteins.

Recent studies revealed that the *Pm4* resistance gene corresponds to previously identified wheat blast resistances *Rmg7* and *Rmg8*, and established that Pm4a, Pm4b and Pm4f can recognize the wheat blast effector AVR-Rmg8 (refs. ^12,13^). AVR-Rmg8 (110 residues) is considerably smaller than AvrPm4 and lacks an RNase-like domain^14^. However, it shares a nearly identical sequence motif with the MEA domain of AvrPm4 (Fig. 1c), suggesting the importance of this domain for Avr recognition by Pm4/Rmg8. Interestingly, we found that the MEA domain of AvrPm4 shows sequence similarity to MED15, a crucial coactivator in eukaryotic transcription, and to EBNA-3B from the Epstein–Barr virus, which is also implicated in transcriptional control (Fig. 1 and Extended Data Fig. 2)^31,32,36^. Additionally, the AvrPm4 MEA domain has a predicted NLS and a high probability of binding DNA (Extended Data Fig. 2d,e). Future experiments should therefore explore whether the MEA domain plays a role in transcriptional manipulation of the host, which may contribute to *B. g. tritici* virulence. *AvrPm4* and *AVR-Rmg8* are conserved within the gene pools of *B. g. tritici* and wheat blast, respectively, indicating an important role of these effectors in pathogen fitness. Considering that the MEA domain might also be implicated in Avr recognition, as suggested by the nearly identical sequence motifs shared by AvrPm4 and AVR-Rmg8, this might represent an important trade-off between preservation of effector function and evasion of Pm4 recognition for the fungal pathogen.

Mutation or loss of Avr effectors is a common mechanism to evade *R*-gene-mediated immunity in fungal plant pathogens^37^. The loss of Avr effectors with crucial virulence functions might, however, result in reduced pathogen fitness. Multiple fungal plant pathogens therefore secrete suppressor proteins to mask Avr effectors or suppress NLR-mediated immunity, thereby allowing them to retain important Avr effectors^24^. Previous studies in *B. g. tritici* identified SvrPm3, an RNase-like effector that suppresses the recognition of AvrPm3 effectors by NLR-type Pm3 resistance proteins^19,27^. Like SvrPm3, the SvrPm4 effector belongs to the group of RNase-like effectors. Our study reveals that a member of this large effector group suppresses recognition by a KFP immune receptor. This underscores the diversity of molecular activities of these effectors and their importance as suppressors of race-specific resistance. Intriguingly, a study in the blast pathogen (*Magnaporthe* spp.) has identified the *PWT4* effector, naturally present in oat-infecting blast isolates, as a suppressor of *Pm4* (*Rmg8*) when expressed in wheat blast^38^. This finding further highlights the similarities in genetic control of avirulence/virulence on *Pm4* between the powdery mildew and blast pathosystems.

Our GWAS and QTL analyses indicate that *SvrPm4* is the main factor controlling virulence on *Pm4a* and *Pm4b* in the global *B. g. tritici* population (Fig. 4 and Extended Data Fig. 9). The importance of the *SvrPm4* locus is further corroborated by the fact that a genetic association between this locus and Pm4 resistance in wheat was also described in a recent study using host–pathogen biGWAS^39^. Importantly, besides the active SvrPm4^JIW2^ and the inactive svrPm4^96224^ variants, we found a staggering array of additional SvrPm4 variants in the *B. g. tritici* population that so far remain untested for their ability to suppress *Pm4*. Phenotype–genotype correlations suggest that additional *SvrPm4* variants may act as active suppressors against *Pm4* (Extended Data Fig. 9), while others might target only a subset of *Pm4* alleles, thereby probably explaining the subtle differences in recognition spectra provided by different *Pm4* alleles^11^ (see also this study).

The *SvrPm4* effector gene (*Bgt-51526*) was previously described as an *Avr* factor of the wheat NLR-encoding gene *Pm1a*^35^. Interestingly, we found that the active suppressor variant SvrPm4^JIW2^ triggers Pm1a-mediated HR in *N. benthamiana*, while the inactive variant SvrPm4^96224^ evades Pm1a recognition (Fig. 5c). This observation suggests that combining *Pm4* and *Pm1a* in the same wheat genotype could provide complementary resistance activities towards *B. g. tritici*. Considering the diversity of the *SvrPm4* gene, it will be important to establish whether the suppression of *Pm4* and the evasion of *Pm1a* recognition are indeed mutually exclusive, which would allow the efficient generation of disruptive selection pressures on this effector through the combination of *Pm4* and *Pm1a* in wheat breeding programmes. We hypothesize that such an approach may prove more robust than *R* gene stacks using independently acting resistance genes and could therefore represent a promising strategy to achieve durable resistance against *B. g. tritici* in wheat.

The GWAS analysis on *Pm4b* revealed a second minor locus residing on chromosome 4. However, the same locus did not show a significant association with virulence on *Pm4a* and was also not identified in our QTL mapping analysis, indicating that it represents a minor or *Pm4b*-specific factor. Future studies should dissect the complex interactions between the largely conserved *AvrPm4*, the large diversity of *SvrPm4* and their consequences for *Pm4* suppression as well as the potential existence of additional minor factors.

The *Pm4* gene exhibits alternative splicing that results in two isoforms, Pm4-V1 and Pm4-V2, which share an identical kinase domain and are both necessary for resistance. Pm4b-V1 localizes to the cytoplasm but relocalizes to the ER membrane upon co-expression with Pm4b-V2 (ref. ^11^). We found that AvrPm4 interacts with both isoforms and relocalizes from the cytoplasm to the ER membrane upon co-expression with Pm4b-V2 (Fig. 3a–d). Furthermore, we found evidence for auto- and transphosphorylation activities of both Pm4 isoforms that result in AvrPm4 phosphorylation (Fig. 3e,f). The importance of Pm4 kinase activity for resistance is further highlighted by the observation that all *Pm4* alleles with documented resistance activity against either *B. g. tritici* or wheat blast (namely, *Pm4a*, *Pm4b* and *Pm4f*) exhibit kinase activity, while the non-functional *Pm4g* allele does not. Strikingly, when investigating the ability of Pm4 to phosphorylate AvrPm4, we also observed phosphorylation of the non-recognized, truncated avrPm4^4AB-2^ variant. This suggests that AvrPm4 phosphorylation alone is not decisive for the initiation of *Pm4*-mediated cell death. This finding contrasts with a recent report that observed phosphorylation of the Avr protein PWT4 in the presence of the KFP RWT4 but did not detect phosphorylation of a non-recognized PWT4 variant^17^. These differences could indicate that molecular resistance mechanisms among KFPs are diverse, similar to the functional diversity observed for NLR-type immune receptors.

The wheat KFPs *Sr62* and *WTK3* were recently found to depend on an NLR protein for HR induction^7,8^. Consistent with the existence of another wheat component, *Pm4* and *AvrPm4* co-expression in the heterologous *N. benthamiana* system failed to trigger HR (Extended Data Fig. 3). We therefore hypothesize that *Pm4*, like other KFPs, relies on another host protein, possibly an executor NLR conserved in the wheat gene pool, for cell death induction. Identifying this unknown component should be a priority of future studies to mechanistically understand the induction of *Pm4*-mediated resistance. Additionally, the striking parallels between *B. g. tritici* and wheat blast pathogens and their Avr and Svr effectors in the context of *Pm4*/*Rmg8* resistance provides the unique opportunity to study the complex molecular events during *Pm4*-mediated immunity in two evolutionarily distant pathosystems.

With a quickly increasing number of identified and characterized R/Avr pairs, in both powdery mildew and wheat blast, a complex molecular network also involving suppressors, modifiers and additional resistance genes has begun to be unravelled. In fact, besides *Pm4*/*Rmg8*/*Rmg7*, recent studies showed further parallels between cereal powdery mildew and wheat blast pathosystems. The wheat *RWT4* resistance gene, identified as a key host-specificity factor against the blast pathogen, is allelic to the *B. g. tritici* resistance gene *Pm24* (ref. ^16^), and the barley powdery mildew resistance gene *MLA3* recognizes the wheat blast effector *PWL2* (ref. ^40^). Moreover, our finding that the active suppressor of Pm4, *SvrPm4*^*JIW2*^, is recognized by the NLR *Pm1a*, while the inactive *svrPm4*^*96224*^ evades recognition, further highlights the complexity of the R–Avr molecular networks even within the wheat powdery mildew pathosystem. In conclusion, our study advocates for a shift from single-gene strategies to resistance gene stacking, combining kinase-based receptors such as *Pm4* with NLRs such as *Pm1a*. We advocate joint wheat breeding programmes for mildew and blast resistance to efficiently use overlaps and synergies between resistance sources, resulting in multilayered and potentially more durable pathogen resistance.

## Methods

### Fungal and plant material and phenotyping experiments

The *B. g. tritici* reference isolate CHE_96224 and the mapping population CHE_96224 × THUN-12 were previously described^34^. The 78 *B. g. tritici* isolates used for virulence phenotyping and GWAS analysis were selected from a worldwide diversity panel^33^. Isolates were maintained clonally on leaf segments of the susceptible wheat cultivar Kanzler (K-57220) placed on food-grade agar (0.5% PanReac AppliChem) supplemented with 4.23 mM benzimidazole (Merck; CAS 51-17-2)^41^.

The NILs Khapli/8*CC/8*Fed (*Pm4a*), Khapli/8*CC (*Pm4a*) and W804/8*Fed (*Pm4b*) were previously described^11^. The *Pm4b*-overexpressing transgenic line (event #52; ref. ^11^) was self-fertilized to the T_4_ generation, producing T_5_ homozygous *Pm4b* transgenic plants. A segregating, transgene-free sister line (S#52) was used as a control. For virulence specificity testing of *B. g. tritici* mutants, NILs with specific resistance genes were used: Axminster/8*Chancellor (*Pm1a*)^42^, Federation*4/Ulka (*Pm2a*)^43^, Asosan/8*Chancellor, Chul/8*Chancellor, Sonora/8*Chancellor, Kolibri and Michigan Amber/8*Chancellor (*Pm3a*, *Pm3b*, *Pm3c*, *Pm3d* and *Pm3f*, respectively)^44^, transgenic Pm3e#2 (*Pm3e*)^45^, Kavkaz/4*Federation (*Pm8*)^46^, USDA_Pm24 (*Pm24*) and “Amigo” (1AL.1RS translocation line, *Pm17*)^47^. Additionally, *A. tauschii* accessions TOWWC087, TOWWC112 and TOWWC154 were included (*WTK4*)^48^.

*B. g. tritici* virulence phenotyping was performed using the primary leaf of 8–12-day-old wheat seedlings, grown under 16 h light/8 h dark cycles at 18 °C and 60% humidity. The leaves were cut into 3-cm fragments and placed on Petri dishes with 0.5% water-agar containing 4.23 mM benzimidazole (Merck; CAS 51-17-2). Spores were collected with a funnel and blow-inoculated onto the Petri dish. The phenotype was then evaluated by eye as the percentage of leaf coverage by sporulating colonies (0–100%) after six to eight days.

### Mutagenesis and mutant analyses

*B. g. tritici* mutants were isolated as described previously^22^. Briefly, spores of the avirulent isolate CHE_96224 were irradiated with UV light and propagated on cv. Kanzler three times, before being used for infecting *Pm4a*- and *Pm4b*-containing NILs (see above). Colonies growing on *Pm4a* or *Pm4b* NILs were isolated from single spores, and their virulence phenotype was confirmed via an infection test. Finally, spores were collected, frozen in liquid nitrogen and ground using a tissue homogenizer, and DNA was extracted using a modified CTAB method (as described previously^19^). Library preparation and Illumina sequencing were performed at the Functional Genomics Center Zurich (Zurich, Switzerland) and Novogene (Cambridge, UK) using NovaSeq 6000 technology, as described previously^22^.

The raw reads obtained were trimmed using Trimmomatic v.0.39 (ref. ^49^), then mapped to the reference genome of the *B. g. tritici* isolate CHE_96224, v.3.16 (ref. ^34^) using bwa mem v.0.7 (ref. ^50^). The last steps involved sorting, removing duplicates and indexing the bam files, using Samtools v.1.9 (ref. ^51^). Haplotype calling, transposable element insertions and gene deletions or duplications were performed as described previously^22^. As performed in our previous study, variants less than 1.5 or 2 kb away (for SNPs and transposable element insertions, respectively) from annotated genes were considered for further investigations (Supplementary Tables 1 and 2).

### Confocal imaging

Live-cell imaging was performed using a Stellaris 5 inverted confocal laser scanning microscope (Leica Microsystems) equipped with an external fluorescence light source (Lumencor LED3), a 405-nm diode laser, and a Leica white light laser. Confocal imaging was conducted as previously described^11^, with minor modifications. Briefly, four-week-old *N. benthamiana* plants were infiltrated with *Agrobacterium tumefaciens* carrying the plasmids of interest. Three days post infiltration, 5 mm × 5 mm leaf samples were mounted between a glass slide and a coverslip in a drop of water. Fluorescence was observed using the following excitation and emission settings: mTurquoise, excitation at 405 nm, emission collected between 425 and 522 nm; Venus, excitation at 515 nm, emission collected between 520 and 550 nm; mCherry, excitation at 587 nm, emission collected between 592 and 652 nm. Fluorescence intensities in the cytosol and ER were measured using the Plot Line plugin in Fiji (https://fiji.sc/). All experiments were conducted under strictly identical confocal acquisition parameters, including laser power, gain, zoom factor, resolution and emission detection settings, ensuring minimal background noise and avoiding pixel saturation. Pseudo-coloured images were generated using the ‘Green’, ‘Magenta’ and ‘Turquoise’ look-up tables in Fiji.

### Cloning of expression constructs

The coding sequences of *AvrPm4*^*96224*^, *avrPm4*^*4AB-2*^, *avrPm4*^*4AB-6*^, *SvrPm4*^*JIW2*^, *SvrPm4*^*96224*^ and *BgtE-5764*^*96224*^ lacking the signal peptide (as detected by SignalP4.0; ref. ^52^), were codon-optimized for wheat expression using the codon-optimization tool from Integrated DNA Technologies (https://eu.idtdna.com) and subsequently gene-synthesized by our commercial partner BioCat GmbH (https://www.biocat.com). Codon-optimized *SvrPm4* variants were cloned into the Gateway-compatible pENTR plasmid using In-Fusion cloning (Takara Bio) and then mobilized into the binary expression vector pIPKb004 (ref. ^53^) using LR Clonase II (Invitrogen). The pIPKb004-Pm1a-HA construct has been previously described^42^. *AvrPm4*^*96224*^, *avrPm4*^*4AB-2*^, *BgtE-5764*^*96224*^ and *WTK4* were cloned into the Gateway-compatible pDONR207 plasmid and then into the destination vectors for the split-luciferase assay (GW_NLUC, NLUC_GW, GW_CLUC and CLUC_GW^54^) using LR Clonase II (Invitrogen); LUC-tagged Pm4b-V1 and Pm4b-V2 have been previously described^11^. For wheat protoplast expression, all previously described constructs were cloned into the pTA22 vector^55^. Additionally, *avrPm4*^*4AB-6*^, *AvrSr50* (ref. ^56^), *AvrPm3*^*a2/f2*^ (ref. ^19^), *Pm4b-V1*, *Pm4b-V2*, and *GUS* were PCR amplified, cloned into the pDONR207 vector and subsequently transferred into the binary expression vector pTA22 using LR Clonase II (Invitrogen). *YFP-pTA22* was used as described previously^55^.

The coding sequence for *mTurquoise-AvrPm4*^*96224*^ was codon-optimized for plant expression using the optimization tools provided by Integrated DNA Technologies (https://eu.idtdna.com). Synthesis of *mTurquoise-AvrPm4*^*96224*^, *mCherry-Pm4b-V1* and *Venus-Pm4b-V2* as well as cloning into the Gateway-compatible vector pDONR221 was performed by Life Technologies Europe BV (Thermo Fisher Scientific). These constructs were subsequently transferred into the binary expression vector pIPKb004 (ref. ^53^) using LR Clonase II enzyme mix (Invitrogen).

The coding sequences of *Pm4b-V1*, *Pm4b-V1*^*D170N*^, *Pm4b-V1*^*D188N*^, *Pm4a-V1*, *Pm4f-V1* and *Pm4g-V1* (full-length variants), as well as *Pm4b-V2ΔTMD*, *Pm4b-V2ΔTMD*^*D170N*^, *Pm4b-V2ΔTMD*^*D188N*^, *Pm4a-V2*, *Pm4f-V2*, *Pm4g-V2* (kinase + C2D domain), *AvrPm4*^*96224*^ and *avrPm4*^*AB22*^, were PCR amplified and cloned in frame into the vector pMAL-C4E between the restriction sites EcoRI and BamHI, to generate fusion proteins carrying an N-terminal MBP tag^57^. Ligation of the PCR product and vector backbone was performed using T4 DNA Ligase (New England Biolabs). The His–MBP Exon 1–5 coding sequence was PCR amplified using primers PC_90 and PC_91 with flanking BsaI recognition sites and unique overhangs to enable directional and seamless assembly. The construct was assembled using the Golden Gate cloning system, using type IIS restriction enzymes and T4 DNA ligase in a one-tube reaction. The assembled product was cloned into the destination vector pET28a(+)-GG.

The constructs were verified via full plasmid sequencing (Microsynth AG). The primers used for cloning are listed in Supplementary Table 4. All codon-optimized effector and resistance gene sequences used in this study are listed in Supplementary Table 5. All expression constructs were transformed into the *A. tumefaciens* strain GV3101 via electroporation.

### Cell death assay in wheat protoplasts

For the cell death assay in wheat protoplasts, we followed the description in a previous study^55^. Briefly, *Avr* genes and *R* genes were cloned into the plasmid pTA22 without tags and subsequently extracted using the Xtra Midi Plus Endotoxin-free MidiPrep Kit (Macherey-Nagel). The DNA was eluted in ultra-pure water. Transgenic seedlings of susceptible cultivar Bobwhite S26 overexpressing Pm4b (#52) and its respective sister line (S#52) were grown in a chamber under a cycle of 12 h light (100 µmol m^−2^ s^−1^) and 12 h dark at 24 °C for seven days. The protoplasts were isolated by peeling off the epidermis and digesting the leaf in a cellulase RS and macerozyme R-10 (Onozuka; Yakult Honsha) solution (enzyme solution: 20 mM MES-KOH, 0.6 M mannitol, 10 mM KCl, Cellulase RS 1.5% (w/v), Macerozyme R-10 0.75% (w/v), 10 mM CaCl_2_, bovine serum albumin (BSA) 0.1% (w/v)). After the purification steps, performed as previously described^55^, the protoplast solution was diluted to a concentration of 3 × 105 cells per millilitre. The different plasmids were mixed for transfection in a 2-ml tube. Three picomoles (3 pmol) of the plasmids containing *YFP* and the individual *Avr*s were used for transfection. Due to Pm4b-V2 autoactivity and to maintain an equal ratio between Pm4b-V1 and Pm4b-V2, 0.5 pmol of plasmids containing *Pm4b-V1*, *Pm4b-V1*^*D188N*^, *Pm4b-V2* and *Pm4b-V2*^*D188N*^ were used. Where one DNA component is missing, the amount of DNA was compensated with a GUS-expressing construct. Two hundred microlitres of protoplasts were then added to the DNA together with a PEG solution (volume PEG solution, 200 μl; volume of DNA mix, ~230 μl; 40% w/v PEG-4000, 0.2 M mannitol, 100 mM CaCl_2_). After a gentle homogenization, the transfection reaction was stopped by adding W5 solution (2 mM MES-KOH, 5 mM KCl, 125 mM CaCl_2_, 154 mM NaCl). All the reagents used for preparing the solutions were from Sigma-Aldrich. After the protoplasts were transferred into a 12-well cell culture plate, they were incubated at 23 °C for 16–20 h in the dark. The next day, YFP fluorescence was measured (excitation, 500 nm; emission, 541 nm) with two technical replicates and three biological replicates per treatment using a microplate reader (Synergy H1, BioTek Instruments). Each experiment was repeated two to three times as indicated in the figure legends. All the measurements were normalized to the average of values from the GUS-transfected protoplasts for each experiment individually.

### *Agrobacterium*-mediated gene expression in *N. benthamiana*

*Agrobacterium*-mediated expression in *N. benthamiana* was performed as previously described^27^. In brief, *A. tumefaciens* (strain GV3101) was grown in liquid lysogeny broth (LB) containing appropriate antibiotics overnight at 28 °C. For *N. benthamiana* infiltration, cultures were briefly washed in LB medium and resuspended in infiltration medium (200 µM acetosyringone, 10 mM MgCl_2_, 10 mM MES-KOH pH 5.6) to an OD_600_ of 1.2 for HR testing, and to an OD_600_ of 1 for split-luciferase and colocalization experiments, subsequently incubated at 28 °C for two to four hours and finally infiltrated into leaves of three- to four-week-old *N. benthamiana* plants.

### Split-luciferase assay

All the constructs co-infiltrated were previously mixed in a 1:1:1 ratio with the third component being the p19-silencing-suppressor strain^58^. Transient expression via agroinfiltration in *N. benthamiana* was performed as described above. At three days post infiltration, 6-mm leaf discs were collected from each leaf and incubated in buffer containing 10 mM MES-KOH (pH 5.6) and 10 mM MgCl_2_. Subsequently, 1 mM luciferin (BioVision) in 0.5% DMSO was added, and after 5 min of incubation, luminescence was measured for 25 min (200 ms per well) using the LUMI imaging system (Tecan). The sum of the values per well was then used for statistical analyses. Two technical replicates and at least 16 biological replicates were measured for each treatment, for a total of three experiments.

### Recombinant protein expression, purification and in vitro kinase assays

The N-terminally MBP-tagged constructs in the pMAL-c4E vector^57^ were transformed into BL21 pLYsS chemically competent Rosetta cells. Ten millilitres of an overnight culture was added to 1 l of LB media, the respective antibiotic and 20% glucose to an OD of 0.6 to 0.8. Protein expression was then induced with 300 mM IPTG, and the cells were grown overnight with shaking at a reduced temperature of 18 °C.

After the cells were harvested via centrifugation at 5,000 *g* for 20 min, the pellet was resuspended in 40 ml of purification buffer (50 mM HEPES-KOH (pH 7.2), 5% glycerol and 1x protease inhibitor tablets (cOmplete EDTA-free; Roche)) and lysed via sonication (4 × 20 s with 40-s breaks at high intensity). Next, the lysed cells were centrifuged at 35,000 *g* for 30 min at 4 °C. The supernatant was further incubated with washed amylose resin (New England Biolabs) and an addition of 2 mM DTT and 300 mM NaCl. After 30 min of incubation at 4 °C on an overhead rotation system, the resin was collected via centrifugation at 1,000 *g* for 5 min at 4 °C. The extraction was then washed four times with a wash buffer (50 mM HEPES-KOH (pH 7.2), 5% glycerol and 300 mM NaCl, 1x protease inhibitor cocktail). The proteins of interest were then eluted using a maltose elution buffer (50 mM HEPES-KOH (pH 7.2), 5% glycerol, 100 mM NaCl and 200 mM maltose, 2 mM DTT). To exchange the buffer in which the proteins were eluted, the samples were run through 50-kDa exclusion centrifuge filters (Amicon, Ultra Zentrifugenfilter, 50 kDa MWCO) and diluted with the elution buffer lacking maltose. MBP–AvrPm4^96224^ (expected size, 80 kDa) migrated as a band at around 110 kDa, with degradation products ranging from 110 to 70 kDa, while MBP–avrPm4^4AB-2^ (expected size, 67 kDa) appeared as a strong band at 85 kDa and a minor band at 70 kDa (Extended Data Fig. 5b). Removal of the MBP tag from the AvrPm4 constructs resulted in insoluble protein. We therefore used tagged AvrPm4 proteins for the experiments (Extended Data Fig. 5b).

Expression and purification of the N-terminally His–MBP-tagged Exon1–5 construct were carried out following a previously described protocol^59^, with some modifications. For this construct, the lysis buffer consisted of 50 mM Tris-HCl (pH 7.5), 200 mM NaCl, 10% glycerol, 5 mM imidazole, 1 mM PMSF and 1× protease inhibitor cocktail (Roche). For the purification procedure, HisPur Cobalt Resin (Thermo Fisher Scientific) was used. The wash buffer contained 50 mM Tris-HCl (pH 7.5), 300 mM NaCl, 10 mM imidazole and 1 mM PMSF. Proteins were eluted from the resin at 4 °C using elution buffer composed of 50 mM Tris-HCl (pH 7.5), 200 mM NaCl, 10% glycerol and 250 mM imidazole. Protein purity and concentration were assessed via SDS–PAGE, using BSA as a standard (loading 1 µg, 0.5 µg, 0.25 µg and 0.1 µg of BSA, respectively). The proteins (samples and BSA standard) were stained with Coomassie Brilliant Blue R-250, and protein abundance was estimated on the basis of band intensity analysis using ImageJ (version 1.54)^60^.

For the in vitro kinase assay, 1 µg of purified protein was incubated in a 20-µl reaction containing 1 µCi [γ-^32^P]ATP, 10 mM MgCl_2_, 10 mM MnCl_2_, 50 mM HEPES-KOH (pH 7.2), 5% glycerol, 100 mM NaCl and 2 mM ATP for 60 min at 37 °C. Proteins were separated via SDS–PAGE and transferred onto a PVDF membrane, which was then exposed overnight to a phosphor screen before imaging using the Amersham Typhoon system (GE Healthcare Life Sciences).

### Protein detection

Immunoblotting was initiated by blocking the membrane with 5% (w/v) non-fat dry milk in TBS-T. MBP-tagged proteins were detected using anti-MBP antibody (New England Biolabs; IgG2a, E8032S) at a 1:3,000 dilution. For LUC-tagged proteins, a polyclonal anti-luciferase antibody (Sigma-Aldrich; L0159) was used at 1:3,000 dilution, followed by a secondary anti-rabbit HRP-conjugated antibody (LabForce; sc-2357), also at 1:3,000 dilution. Detection of mTurquoise- and Venus-tagged proteins was carried out using anti-GFP antibody (clone B-2; Santa Cruz Biotechnology; sc-9996) at a 1:5,000 dilution. mCherry-tagged proteins were detected using anti-RFP antibody (clone 6G6; Chromotek) at a 1:5,000 dilution. In both cases, the corresponding secondary antibody was an anti-mouse HRP-conjugated antibody (Promega; W402B) at 1:10,000 dilution. Peroxidase chemiluminescence was detected using a Fusion FX Imaging System (Vilber Lourmat) after the application of Western Bright ECL HRP substrate (Advansta). The SuperSignal West Femto substrate (Thermo Scientific) was used when maximum sensitivity was required.

### HR measurement in *N. benthamiana*

To assess HR responses upon co-expression of effector and *R* genes, *Agrobacterium* cultures were prepared as described above and mixed in a 4:1 ratio (effector:*R*) prior to infiltration. Four to five days post infiltration, imaging and quantification of HR responses were achieved using a Fusion FX Imaging System (Vilber Lourmat) and ImageJ as previously described^27^.

### Total protein extraction from *N. benthamiana*

*N. benthamiana* tissue for total protein extraction was harvested three days after *Agrobacterium* infiltration by cutting ten leaf disks (6 mm in diameter) from three independent plants and immediately frozen in liquid nitrogen. The samples were collected in a 2-ml tube with three glass beads and next ground using a tissue homogenizer. Then, 1.5× Lämmli buffer was added, and the samples were incubated at 95 °C for 10 min. After 1 min of centrifugation at 16,200 *g*, the supernatant was collected. The proteins in the supernatant were separated via SDS–PAGE and subsequently blotted onto a PVDF or nitrocellulose membrane, which was then subjected to immunoblotting (described above).

### Structural modelling and other bioinformatic analyses

Domain prediction of AvrPm4^96224^ was performed with an NCBI conserved domain search (available through https://www.ncbi.nlm.nih.gov/Structure/cdd/wrpsb.cgi). Modelling of AvrPm4^96224^ was performed using AlphaFold3, available through https://alphafoldserver.com/. Structural alignment of AvrPm4 with AvrPm2 (Extended Data Fig. 2a) was performed with TMalign^61^. The dot plot of AvrPm4^96224^ was visualized using in-house software by T.W. based on the program dotter (available through Ubuntu repositories). Protein alignments were performed with ClustalOmega and visualized using CLC Main Workbench. The NLS was predicted using NLStradamus (http://www.moseslab.csb.utoronto.ca/NLStradamus/). The DNA binding probability was predicted using DP-bind (https://lcg.rit.albany.edu/dp-bind/) with the standard parameters; the raw data are in Supplementary Table 6. Bins of ten amino acid residues containing their average value (between 0 (non-binding) and 1 (binding)) were created and used for producing the density plot depicted in Extended Data Fig. 3d.

### Statistical analyses

All statistical analyses were performed in R/RStudio (v.4.3.1 (ref. ^62^) and v.2023.06.1 (ref. ^63^), respectively). To evaluate differences between treatments in all protoplast and *N. benthamiana* cell death assays, ANOVA followed by Tukey’s HSD test was performed (R package multcomp)^64^. For the split-luciferase assay, an ANOVA followed by multiple comparison of means was performed. To assign letters, the commands glht and cld from the R package multcomp^64^ were used. Package ggplot2 (ref. ^65^) was used for producing the graphs. The raw data are in Supplementary Table 7.

### GWAS analysis

For the GWAS analysis, Illumina resequencing data were mapped against the *B. g. tritici* reference genome (Bgt_genome_v3_16) following a procedure described previously^21^. SNPs were identified using the FreeBayes tool with the command freebayes -p 1 (v.1.3.6)^66^. The identified polymorphic sites were then filtered using VCFtools (v.0.1.16)^67^ with the following specifications: max-alleles, 2; min-alleles; maf, 0.05; max-missing, 0.95; minDP, 8. They were then transformed to hapmap format using a custom Perl script available here: https://github.com/MarionCMueller/Scripts/tree/main/vcf_to_hapmap/. Subsequently, GWAS analysis was conducted with virulence phenotyping data of 78 *B. g. tritici* isolates on NILs Khapli/8*CC (*Pm4a*) and W804/8*Fed (*Pm4b*) (Supplementary Table 3) using GAPIT (v.3)^68^ with the following specifications: PCA.total = 3, model = c(“MLM”).

To estimate the interval underlying a significant association in the GWAS analysis, the pairwise *R*^2^ values between markers on chromosomes 4 and 8 with less than 1 Mbp distance were determined using the VCFtools commands ld-window-bp, 1000000; max-alleles, 2; min-alleles, 2; geno-r2 (v.0.1.16)^67^. Finally, SNPs with an *R*^2^ value greater than 0.6 upon pairwise comparison with the most significantly associated SNPs of the GWAS analysis were used to define the genomic candidate interval.

### QTL mapping

QTL mapping was performed on the basis of the previously described genetic map of the cross between THUN-12 (*B. g. triticale*) and CHE_96224 (*B. g. tritici*)^34,42^, which is available from https://github.com/MarionCMueller. The QTL analysis was performed using the r/qtl package in R (v.1.70)^69^. First, the genetic map was processed using the command jittermap and calc.genoprob(step = 2,error.prob = 0.001), followed by QTL analysis using the command scanone(model = “np”). The significance threshold at *α* = 0.05 was determined via calculating 1,000 permutations with scanone. The 1.5LOD interval was determined using lodint.

### Candidate identification and expression analysis of *SvrPm4* candidates

To identify *SvrPm4* candidate genes, the candidate interval on chromosome 8 was first inspected for spurious annotations. Two gene models were detected, *Bgt-51527* and *Bgt-20620-4*, which lacked either a start or stop codon. These genes were therefore excluded from subsequent analysis. Next, sequence variants of the three candidate effectors located in the interval (*Bgt-51526*, *BgtAcSP-30824* and *BgtAcSP-31023*) were detected in a set of reference isolates (CHE_96224, CHE_94202, GBR_JIW2 and ISR_7) on the basis of resequencing data (Supplementary Table 3).

To accurately determine the expression levels of the three candidate effector genes in the isolates CHE_96224, CHE_94202, GBR_JIW2 and ISR_7, RNA sequencing data from these isolates on the susceptible wheat cultivar Chinese Spring were used at two days post inoculation^21,29^. To quantify transcript abundance, the annotation of CHE_96224 was used (available at https://zenodo.org/records/7018501). To avoid quantification biases, the coding sequences of all gene variants present in the four reference isolates to the CHE_96224 coding sequence annotation file were added. Finally, the new Fasta file was processed using the salmon index command from the salmon package (v.1.4.0)^70^, with the chromosomes of the Bgt_genome_v3_16 assemblies as decoys. Transcripts were quantified using the salmon quant −l A command. Subsequently, expression data across all variants for each isolate were merged in R, and RPKM values were calculated using the rpkm function from the package edgeR (v.4.0.16)^71^.

### Reporting summary

Further information on research design is available in the Nature Portfolio Reporting Summary linked to this article.

## Supplementary information

Reporting Summary

Supplementary Tables 1–7 The Supplementary Tables are provided in Excel format. The first sheet (Title_and_Info_Sheet) lists the file contents and table titles. Seven supplementary tables are presented in separate sheets.

## Source data

Source Data Fig. 3 Unprocessed kinase assay blots for Fig. 3e,f (Coomassie-stained membranes and autoradiograph images).

Source Data Extended Data Fig. 4 Unprocessed blots for Extended Data Fig. 4a,c (immunoblots and Ponceau- and Coomassie-stained membranes).

Source Data Extended Data Fig. 5 Unprocessed blots for Extended Data Fig. 5a,b (Coomassie-stained membrane, autoradiograph image and Coomassie-stained gel).

## Data Availability

Raw FASTA sequences of all *B. g. tritici* mutants generated in this study are available through NCBI (BioProject ID: PRJNA1016363). Sequences of the *B. g. tritici* isolates used for GWAS were previously described^33^ and are available through NCBI (PRJNA625429, SRP062198). The *B. g. tritici* isolates used in this study are maintained at the University of Zurich and are available upon request. However, as the isolates may die over time, their long-term accessibility cannot be guaranteed. Source data are provided with this paper. All remaining data are provided in the main text or the supplementary materials.

## Code availability

The Perl script used for converting VCF to HapMap is available at: https://github.com/MarionCMueller/Scripts/tree/main/vcf_to_hapmap/. Scripts and input files used for QTL mapping are available at: https://github.com/MarionCMueller. Other code is available through the Ubuntu software repositories.
