# Supplementary figures and images for: Virulence on Pm4 kinase-based resistance is determined by two divergent wheat powdery mildew effectors
Source: Nat Plants. 2026 Jan 12;12(1):164–78. doi: 10.1038/s41477-025-02180-w (PMC12830362; doi:10.1038/s41477-025-02180-w)

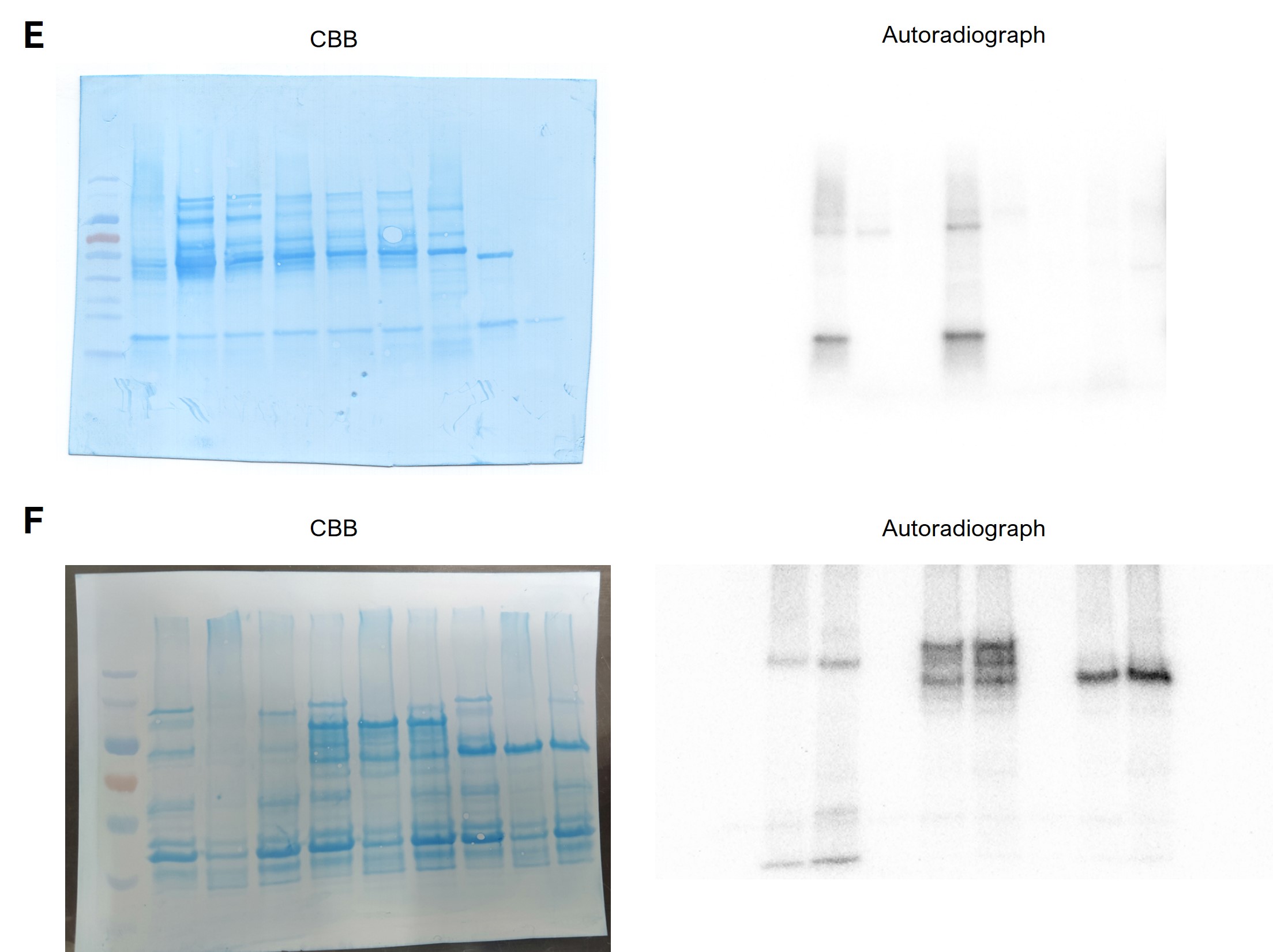

Supplement: Supplementary file 3 — Unprocessed kinase assay blots for Fig. 3e,f (Coomassie-stained membranes and autoradiograph images). [file 41477_2025_2180_MOESM3_ESM.jpg]

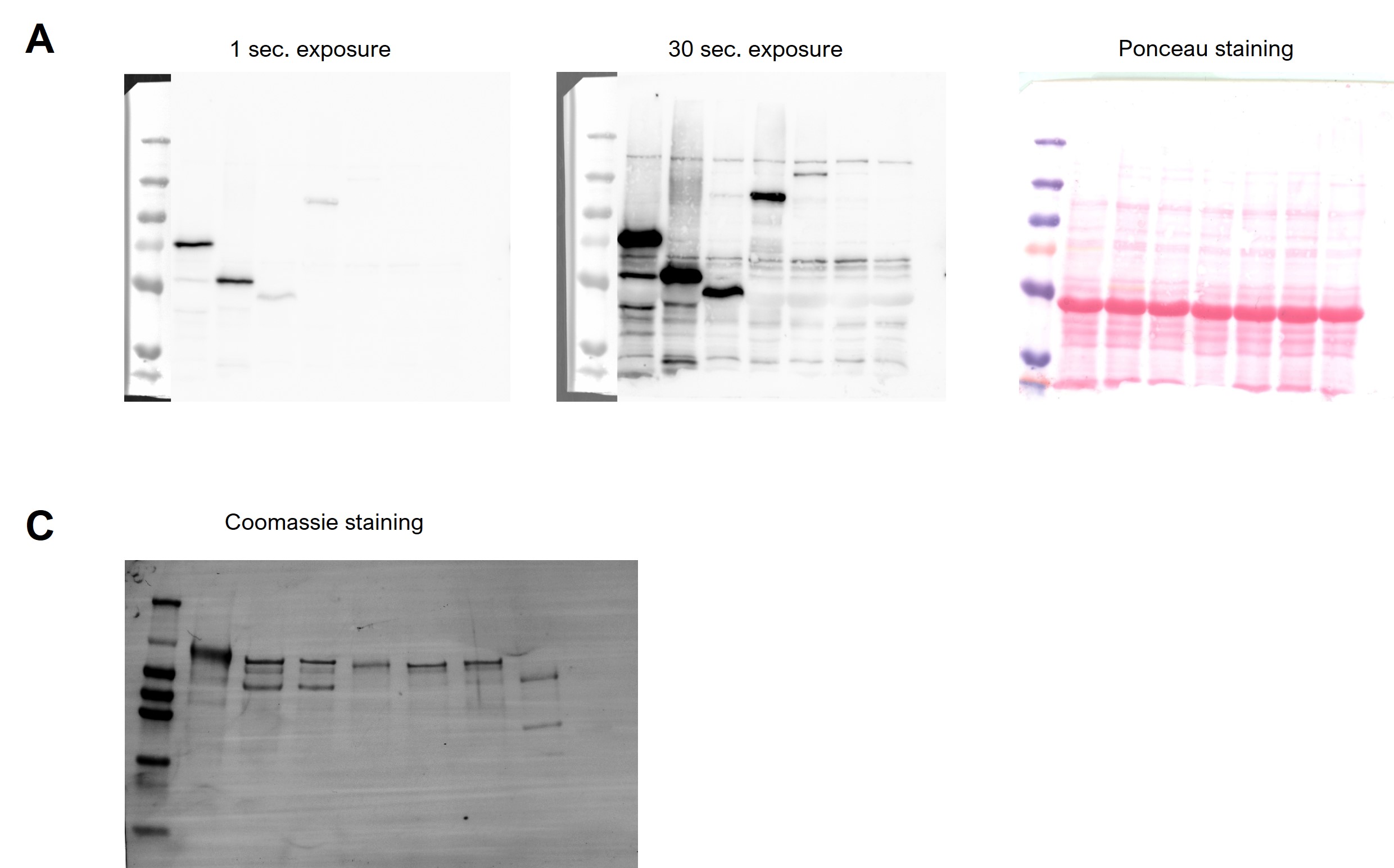

Supplement: Supplementary file 4 — Unprocessed blots for Extended Data Fig. 4a,c (immunoblots and Ponceau- and Coomassie-stained membranes). [file 41477_2025_2180_MOESM4_ESM.jpg]

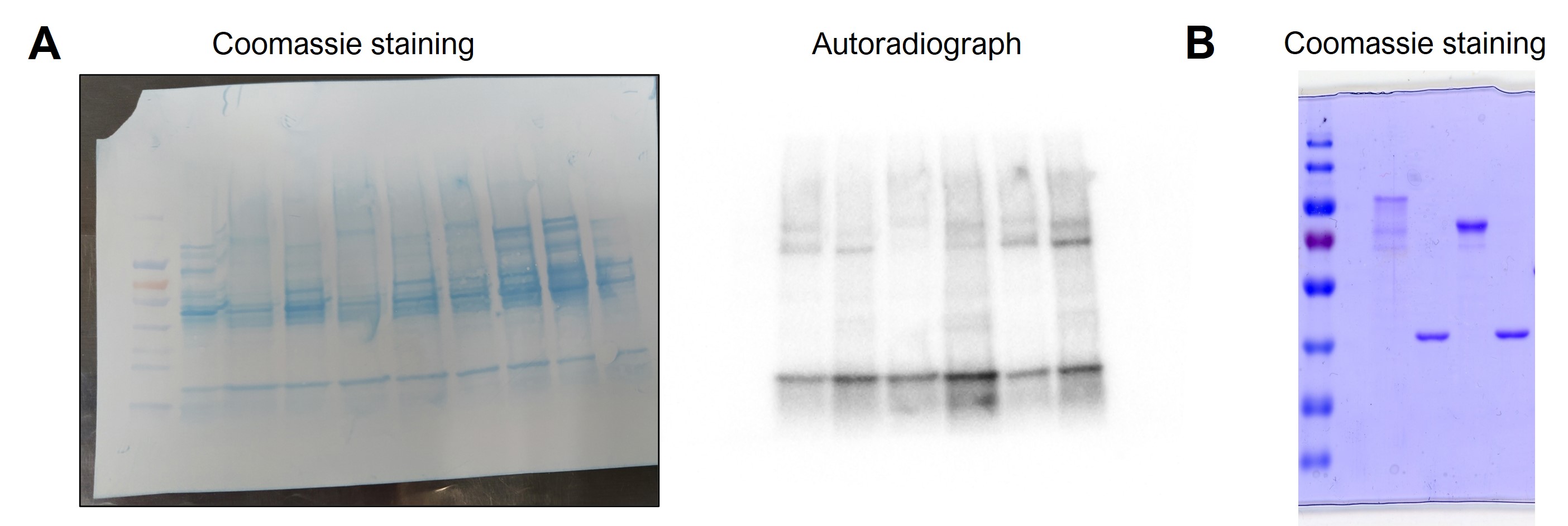

Supplement: Supplementary file 5 — Unprocessed blots for Extended Data Fig. 5a,b (Coomassie-stained membrane, autoradiograph image and Coomassie-stained gel). [file 41477_2025_2180_MOESM5_ESM.jpg]
